# Identification and Evaluations of Novel Insecticidal Proteins from Plants of the Class Polypodiopsida for Crop Protection against Key Lepidopteran Pests

**DOI:** 10.3390/toxins11070383

**Published:** 2019-07-01

**Authors:** Lu Liu, Eric Schepers, Amy Lum, Janet Rice, Nasser Yalpani, Ryan Gerber, Nuria Jiménez-Juárez, Fikru Haile, Alejandra Pascual, Jennifer Barry, Xiuli Qi, Adane Kassa, Matthew J. Heckert, Weiping Xie, Changkui Ding, Jarred Oral, Minh Nguyen, James Le, Lisa Procyk, Scott H. Diehn, Virginia C. Crane, Howard Damude, Carol Pilcher, Russ Booth, Lu Liu, Genhai Zhu, Timothy M. Nowatzki, Mark E. Nelson, Albert L. Lu, Gusui Wu

**Affiliations:** 1 Corteva Agriscience, Hayward, CA 94545, USA; 2 Corteva Agriscience, Johnston, IA 50131, USA

**Keywords:** plant protein, insecticidal, genetically modified, lepidopteran, resistance management

## Abstract

Various lepidopteran insects are responsible for major crop losses worldwide. Although crop plant varieties developed to express *Bacillus thuringiensis* (Bt) proteins are effective at controlling damage from key lepidopteran pests, some insect populations have evolved to be insensitive to certain Bt proteins. Here, we report the discovery of a family of homologous proteins, two of which we have designated IPD083Aa and IPD083Cb, which are from *Adiantum spp*. Both proteins share no known peptide domains, sequence motifs, or signatures with other proteins. Transgenic soybean or corn plants expressing either IPD083Aa or IPD083Cb, respectively, show protection from feeding damage by several key pests under field conditions. The results from comparative studies with major Bt proteins currently deployed in transgenic crops indicate that the IPD083 proteins function by binding to different target sites. These results indicate that IPD083Aa and IPD083Cb can serve as alternatives to traditional Bt-based insect control traits with potential to counter insect resistance to Bt proteins.

## 1. Introduction

Many lepidopteran insect species are economically important pests of crops worldwide. The loss caused by these insects can be billions of US dollars annually and continues to grow [1,2]. The feeding damage caused by corn earworm (CEW, *Helicoverpa zea*) and fall armyworm (FAW, *Spodoptera frugiperda*)), which are primary insect pests of corn (*Zea mays*), and soybean looper (SBL, *Chrysodeixis includens*), velvetbean caterpillar (VBC, *Anticarsia gemmatalis*) and CEW which are primary insect pests of soybean (*Glycine max*), results in reduced crop yield and seed quality [3,4]. *Bacillus thuringiensis* (Bt) crystalline proteins (Cry) such as Cry1Ab, Cry1Fa, and Cry2Ab or vegetative insecticidal proteins (Vip) such as Vip3Aa have been very effective against lepidopteran pests in transgenic corn, soybean, and cotton (*Gossypium hirsutum*) [5,6]. Early transgenic crops deployed for insect control contained Bt proteins functioning with only a single mode of action (MoA) for each of the key pests [7,8,9]. Pyramiding two or more Bt proteins with distinct target sites (for dual or multi-MoA) against each pest has been a useful approach for extending the durability of Bt technology. However, the evolution of insect resistance to these Bt proteins, when previously deployed as a single MoA, can limit the durability improvements realized from this strategy [8,9]. In fact, a survey of recently published literature on insect resistance to Bt crops [10] showed that practical resistance was observed for five out of seven Bt proteins targeting the key lepidopteran species in cotton and corn. Practical resistance was defined as when >50% of a pest population was resistant (survive exposure to concentrations that kill nearly all susceptible individuals) and the efficacy of the Bt crop was reduced in the field [11]. Among major lepidopteran insect species, the FAW is a voracious pest with a wide host range [12]. This migratory insect is found in Neotropical America and in the southern United States [12]. The recent invasion of FAW into Africa and Asia is expected to be a significant threat to several important crops in a region where food security is an important issue [13,14,15]. The FAW is a highly adaptive pest and field-evolved resistance to Cry1Fa, one of the few Bt proteins with effective control against FAW, has been well-documented [10,16]. There is a need to find insecticidal proteins that function through new target sites to support the development of future generations of transgenic insect control crops. Though Bt diversity remains a major source for insecticidal proteins, it has become challenging to find new Bt proteins with an optimal activity spectrum and potency that are suitable to replace those active in current commercial products. Alternative sources, including plants, that produce insecticidal proteins with activity against lepidopteran species, have not been explored extensively. To date, only two major groups of plant-derived proteins - proteinase inhibitors and carbohydrate-binding lectins - have been shown to possess insect inhibiting activity, but their use in transgenic crop plants has had only limited success, especially against lepidopteran pests of major crops [17,18]. However, recent reports on the identification of several insecticidal proteins from non-Bt bacteria for controlling the coleopteran insect western corn rootworm (WCRW; *Diabrotica virgifera virgifera*) in corn and a chitinase-like protein from a plant species for protecting cotton plants from the hemipteran insect whitefly (*Bemisia tabaci*) illustrate the potential of non-Bt sources for new insecticidal protein discovery [19,20,21,22,23].

Here, we report the activity-based discovery and isolation of novel plant-derived proteins from related *Adiantum spp*. Cloning and expression of the proteins in transgenic plants show their ability to provide robust protection from leaf damage caused by major insect pests of corn and soybean. Testing against Bt-resistant insect populations along with competition binding studies revealed that these proteins do not bind to the same target sites that are utilized by the Bt toxins that are expressed in current commercial traits. Interestingly, direct competitive binding studies of these homologous proteins showed that they bind to distinct target sites. These results highlight the potential of plants as rich sources of proteins to be developed for future generations of insect control traits.

## 2. Results

### 2.1. Plant Diversity Screening, Protein Purification, and Identification

**Figure 1:**
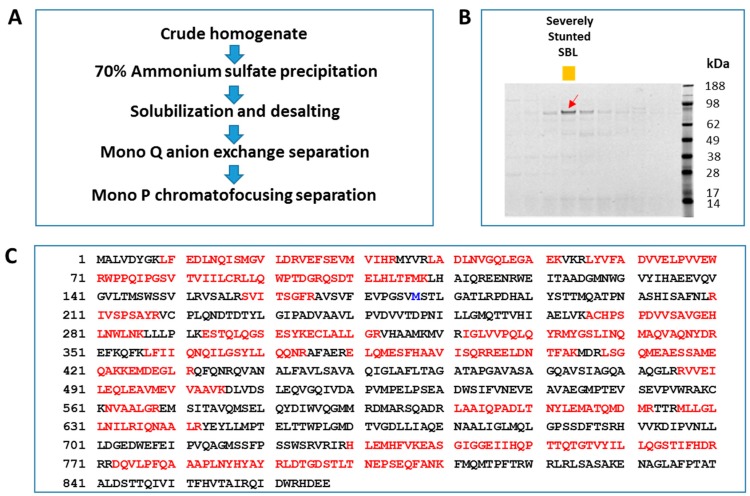
Protein purification process and candidate protein identification by liquid chromatography-tandem mass spectrometry (LC-MS/MS). (**A**) Purification process depicted in the flow-chart was used to purify the IPD083Aa candidate protein from the crude lysate of *A. pedatum* fronds (see Section 4.3). (**B**) Fractions near the active peak after Mono P chromatofocusing separation are shown on a Coomassie blue stained SDS-PAGE gel. The fraction marked with a red arrow showed severe stunting (>60% growth reduction to diet control) to SBL and the corresponding gel band was subjected to in-gel tryptic digestion for LC/MS/MS protein sequence identification. (**C**) Insecticidal protein candidate was identified by searching the LC-MS/MS data of tryptic peptides of the protein band marked in (**B**) against a protein database including the proteins translated from the transcriptome sequence library of the source plant using the Mascot search engine [25]. Sequences in red were matched by tryptic peptide masses of the digested gel band with an overall coverage of 46%. Amino acid residue M177 from the assembled contig was changed into R177 after sequence verification of the RT-PCR product. Mascot research of the corrected sequence improved the coverage to 50% as the peptide fragment from A166 to L209 was matched.

To assess the potential of plant sources for new insecticidal protein discovery with focus on insect pests of major crops, we generated crude protein extracts from vendor-supplied non-biased plant samples and tested them for activity against multiple lepidopteran species using artificial diet bioassays. An extract from one plant sample classified as *Adiantum pedatum* [24] showed insecticidal activity against the larvae of SBL, FAW, and CEW. A transcriptome sequence library of this plant species was generated using Illumina Genome Analyzer IIX and various sequence assembling software packages described in Section 4.2. The assembled sequences in the library were annotated by a homology-based comparison of predicted open reading frames (ORFs) and the public protein sequence database. To identify the protein component(s) responsible for the insecticidal activity, we developed a multi-step chromatography protocol that allowed enrichment of the active protein. Analysis of fractions from the final purification step by SDS-PAGE revealed a major band at ~90 kDa, whose abundance correlated with insecticidal activity (Figure 1A,B). This band was digested with trypsin and subjected to analysis by liquid chromatography-tandem mass spectrometry (LC-MS/MS). We used the LC-MS/MS data of tryptic peptides to search against a protein database which included protein sequences translated from the transcriptome sequence library of this specific *A. pedatum* species using the Mascot search engine [25]. A high confidence match with 46% protein sequence coverage to a hypothetical protein of 867 amino acids was identified, which we designated *IPD083Aa* (GenBank accession number KY558369) (Figure 1C).

### 2.2. Insecticidal Activity Confirmation

**Figure 2:**
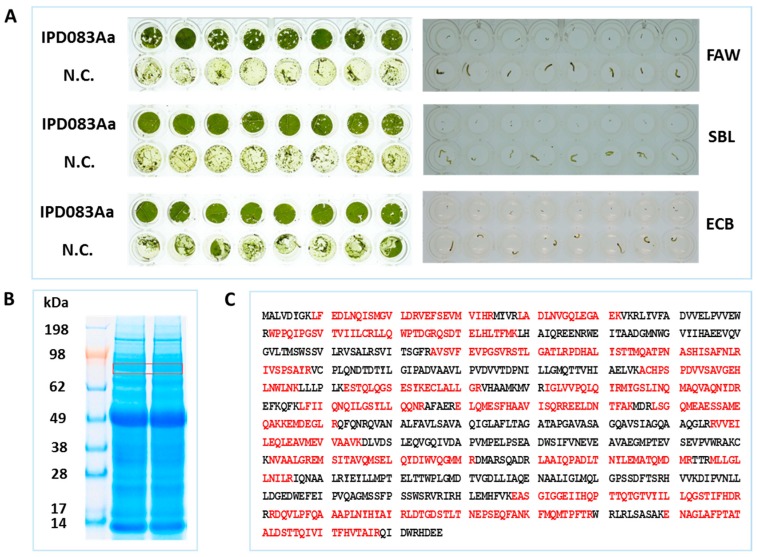
Confirmation of insecticidal activity of IPD083Aa in the common bean transient expression system. (**A**) Images of leaf disk feeding protection of the common bean plant expressing the IPD083Aa protein (left) and the neonates recovered after the bioassay (right). Mortality and/or minimal growth were observed for all neonates recovered from the IPD083Aa expressing leaf disks, while the ones recovered from the vector-only negative control (N.C.) were all alive and much larger in size. (**B**) SDS-PAGE gel image of the crude extract of common bean leaf disks expressing IPD083Aa. Gel band nearby 90 kDa was excised for tryptic digestion and liquid chromatography-tandem mass spectrometry (LC-MS/MS) analysis to confirm the IPD083Aa expression. (**C**) Confirmation of IPD083Aa expression by LC-MS/MS analysis. Sequences in red were matched by tryptic peptide masses of IPD083Aa with an overall coverage of 52%.

We attempted to express the IPD083Aa protein in *Escherichia coli*, but it only produced an insoluble protein. We then expressed the IPD083Aa protein through an *Agrobacterium*–mediated transient expression system in common bean (*Phaseolus vulgaris*) [26,27] to confirm its insecticidal activity. Leaf disks expressing IPD083Aa under the control of a double-enhanced Mirabilis Mosaic Virus (DMMV) promoter [28] showed a clear reduction in feeding damage against FAW, SBL, and the European corn borer (ECB, *Ostrinia nubilalis*) (Figure 2A). In addition, based on our observations of insect movement and growth inhibition (size) for each insect compared to negative control treatments, the data indicate that the limited feeding observed on the common bean leaf disk expressing IPD083Aa caused mortality (Figure 2A). Transient expression of IPD083Aa in the leaf disks was confirmed using LC-MS/MS (Figure 1B,C).

### 2.3. Homolog Identification, Insecticidal Activity, and Spectrum Assessment

**Figure 3:**
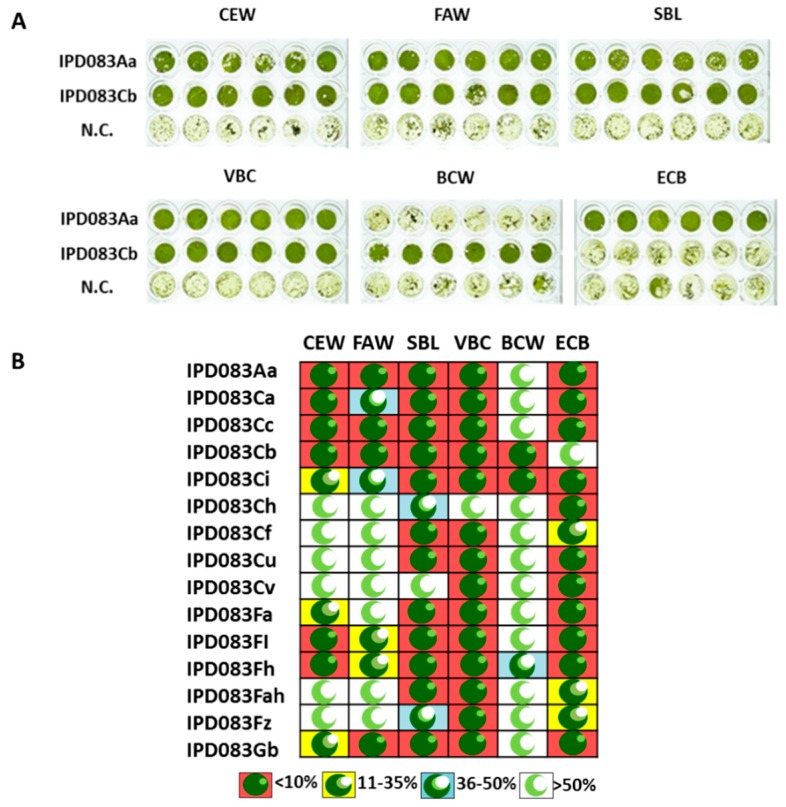
Transient leaf disk feeding damage and activity spectra of selected IPD083 family members. (**A**) Examples of insect leaf disk feeding assays. Images of common bean leaf disks expressing IPD083 homologs were used to estimate the feeding consumption by various lepidopteran species. Final estimation of feeding consumption for each insect was made with at least 12 leaf disks. Leaf disks expressing an empty vector were used as a negative control (N.C.). (**B**) Graphical representations for leaf disks transiently expressing corresponding IPD083 family members shown in Table 1 in common bean with various lepidopteran species. Average feeding damage is shown as the percent of leaf area consumed by insects based on at least six leaf disk replicates. Consumption of more than 50% is considered very weak or no activity for a given IPD083 family member against a given insect.

The observation of insecticidal activity of the IPD083Aa protein prompted us to investigate whether there are homologs of IPD083Aa in other plant species that would have similar properties. As a part of these investigations, we obtained the transcriptome sequence libraries of *A. pedatum*-related plant species that belong to the class Polypodiopsida/Pteridopsida (see Table 1 for selected examples). In addition, screening of the crude lysates showed that most of those plant species also demonstrated insecticidal activity against at least one of the screened pests. A search for homologous proteins using the BLASTp alignment tool [29] against public protein databases and Corteva Argriscience proprietary transcriptome sequence libraries of plant species resulted in the identification of many IPD083Aa homologs (called the “IPD083 family” hereafter), with protein sequence identities ranging from 30% to greater than 90%. So far, the presence of this family of proteins has been found in plant species that belong to the class Polypodiopsida/Pteridopsida. We devised a naming convention to show the protein sequence identity relationship to IPD083Aa. For example, IPD083B, IPD083C, and IPD083D are grouped into brackets that are, 80–89%, 70–79%, and 60–69% identical, respectively, to IPD083Aa at the amino acid sequence level. For homologs within the same bracket of percent identity, a lowercase letter is added based on the chronological order of sequence discovery (e.g., IPD083Ca, IPD083Cb, IPD083Cc, etc.). In cases where more than 26 homologs with >90% homology within the same bracket were identified, two lowercase letters were added, e.g., IPD083Fah was the 33^rd^ homolog of IPD083Fa identified. Analyses of the primary sequences of IPD083Aa and its homologs with protein prediction tools available within InterProScan (comprising PROSITE, PRINTS, Pfam, ProDom, SMART, TIGRFAMs, PIR SuperFamilies) [30] did not reveal any known peptide motifs, domains, or sequence signatures. Homologs selected based on sequence divergence (Table 1) were evaluated for insecticidal activity using the common bean transient expression system. Pairwise protein sequence identities among those homologs are listed in Table S1. Homologs such as IPD083Cc and IPD083Cb are 70 and 71% identical to IPD083Aa, respectively, but they are only 76% identical to each other. As was done for IPD083Aa, its homologs were tested for their ability to protect against feeding damage caused by various lepidopteran species following transient expression in common bean. The levels of damage showed a clear reduction in feeding by CEW, FAW, SBL, VBC, black cutworm (BCW, *Agrotis ipsilon*), and ECB (Figure 3). The results of these assays reveal that homologs within the IPD083 family possess different levels and spectra of insecticidal activity (Figure 3B). IPD083Aa and a homolog, IPD083Cb, were selected for further evaluations in soybean and corn, respectively.

### 2.4. Evaluation of Insecticidal Activity of IPD083Aa in Soybean Plants

**Figure 4:**
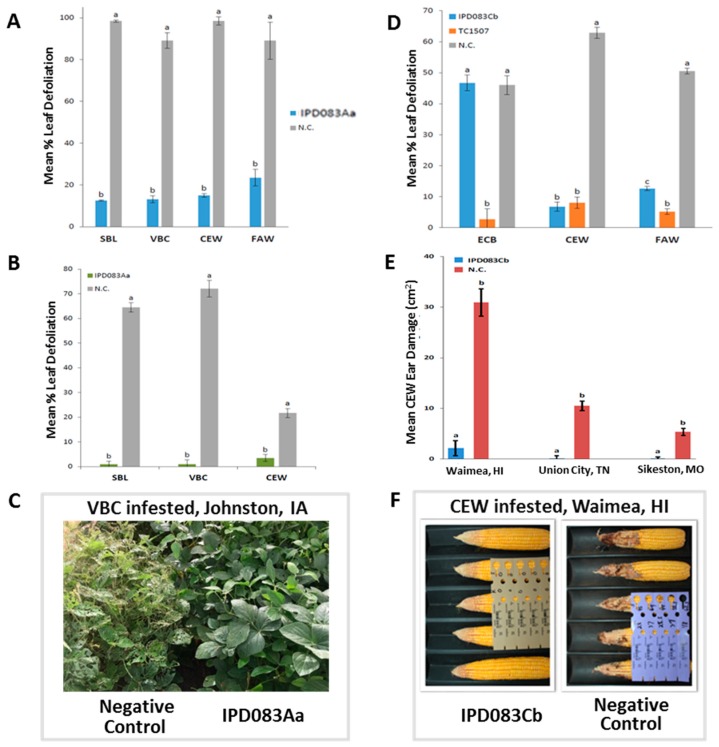
IPD083Aa and IPD083Cb provide protection against insect damage in soybean and corn. (**A**) Leaf disks from T1 homozygous transgenic soybean plants, expressing the IPD083Aa protein, exhibited reduced feeding damage from VBC, SBL, CEW, and FAW. Blue bars represent transgenic soybean plants and gray bars are non-transgenic negative controls (N.C.). A sample size of 40 leaf disks was used for each insect. (**B**) T3 homozygous transgenic soybean plants, expressing the IPD083Aa protein, exhibited a reduction of foliage feeding from VBC, SBL, and CEW in a field testing experiment. Green bars represent transgenic soybean plants and gray bars represent the non-transgenic negative control. The sample size of 80 T3 plants expressing IPD083Aa was used per insect treatment. (**C**) Images of soybean plants with or without the expression of IPD083Aa after VBC infestation in the field. (**D**) Leaf disks from T0 hemizygous transgenic corn plants, expressing the IPD083Cb protein, exhibited reduced feeding damage from CEW and FAW, but not ECB. Blue bars represent IPD083Cb expressing T0 transgenic corn plants, orange bars represent Cry1Fa expressing hemizygous corn plants (TC1507 event) as a positive control, and gray bars represent the non-transgenic negative control. A sample size of 64 leaf disks was used for each insect. (**E**) T2 hybrid transgenic corn plants, expressing the IPD083Cb protein, exhibited ear protection against CEW in fields with different levels of natural CEW infestation. Blue bars represent IPD083Cb-expressing transgenic corn plants and brown bars represent the non-transgenic control. Information on the sample size is listed in Section 4.8. For (**A**), (**B**), (**D**), or (**E**), the results are mean values with standard errors. Bars with different letters differ significantly at the *p* < 0.05 level (Tukey–Kramer test). (**F**) Corn ear images of transgenic plant expressing IPD083Cb (left) and the non-transgenic negative control (right) after heavy natural CEW infestation in a field in Waimea, HI, USA.

To assess IPD083Aa’s potential for protecting soybean plants from SBL, VBC, and CEW damage in plants, transgenic plants were generated by the particle gun bombardment of soybean line TB5 [31] using plasmid vector GmIPD083Aa, containing an expression cassette of the *IPD083Aa* gene under the control of a constitutive *Arabidopsis* ubiquitin promoter (AtUBQ10) [32]. The expression cassette insertion copy number for each of the T0 plants generated was estimated by qPCR as described [33]. Ten greenhouse-grown first-generation (T1) homozygous soybean plants derived from two single copy parental (T0) transgenic soybean plants (called events) expressing IPD083Aa were evaluated for leaf disk feeding protection against neonate SBL, VBC, CEW, and FAW (Figure 4A). The mean consumption of leaf disks across the negative controls ranged from 89 to 98%. In contrast, the mean consumption of IPD083Aa protein-expressing leaf disks was 12.5% (SBL), 13.1% (VBC), 15.0% (CEW), or 23.4% (FAW). In all treatments, insect damage to IPD083Aa protein-expressing plants was significantly lower than for the corresponding negative controls (*p* value < 0.0001). The expression of IPD083Aa in the leaf tissues of five T1 plants was detected by western blot analysis (Figure S1A). In order to have sufficient homozygous seed available for field testing, a seed increase was performed with second-generation (T2) plants derived from efficacious T1 plants. Third-generation (T3) homozygous plants were tested further in a field experiment. Strong overall leaf protection was observed after heavy infestation (1000 pupae/43 m^2^) of SBL or VBC (Figure 4B,C). When challenged with CEW (750 pupae/43 m^2^), soybean plants from the GmIPD083Aa construct also provided protection of leaves (Figure 4B) and pods. These results indicate that expression of the IPD083Aa protein in transgenic soybean plants provides whole-plant protection against its primary lepidopteran pests in the field.

### 2.5. Evaluation of Insecticidal Activity of IPD083Cb in Corn Plants

To evaluate IPD083Cb’s potential for protecting corn plants from CEW and FAW damage, transgenic plants were generated by *Agrobacterium*-mediated transformation [34] using the plasmid vector ZmIPD083Cb containing an expression cassette of the *IPD083Cb* gene under the maize ubiquitin promoter (ZmUBI) [35]. The corn transformation vector backbones expressing IPD083Cb were selected for leaf disk feeding assays with neonate insects. The results showed that IPD083Cb provided feeding protection against CEW and FAW, but not against ECB (Figure 4D), consistent with the activity spectrum observed in the leaf transient expression bioassay (Figure 3). The mean CEW leaf disk feeding consumption for the negative control (63.0%) was much higher than that for the plants expressing IPD083Cb (6.8%), as well as for the commercialized plant event TC1507 expressing Cry1Fa (8.0%), as the laboratory positive control [36]. FAW protection provided by plants expressing IPD083Cb was lower than the positive control (12.6% vs. 5.1% leaf damage), but significantly higher than non-transgenic controls (12.6% vs. 50.0% damage (*p* < 0.0001)). Expression of the IPD083Cb protein in leaf tissue from the plants was confirmed by western blot analysis (Figure S1B). Selected efficacious plants in leaf disk feeding assays were also tested for ear protection (Figure S2). Negative control plants had 13.4 cm^2^ and 10.3 cm^2^ of mean ear damage from CEW (*n* = 7 ears) and FAW (*n* = 19 ears), respectively. In contrast, mean ear damage of TC1507 (positive control) and IPD083Cb plants was comparable for both CEW (*n* = 9 ears) and FAW (*n* = 7 ears). Both showed strong ear protection, with mean ear damage of 1.6 cm^2^ for CEW and 0 cm^2^ for FAW, which was significantly lower than for the negative control ears (*p* < 0.0001). Overall, IPD083Cb provided effective leaf and ear protection from CEW and FAW feeding damage in transgenic corn plants when tested in a greenhouse setting. To further evaluate its efficacy under field conditions, T2 hybrid corn plants derived from eight selected T0 events expressing IPD083Cb were planted at three US locations with varying levels of natural CEW infestations (Table S2). At all three field sites, IPD083Cb provided excellent ear protection from CEW damage, even under a high level of insect pressure (Figure 4E,F).

### 2.6. Recombinant Protein Characterization, Activity, and Spectrum Evaluation

**Figure 5:**
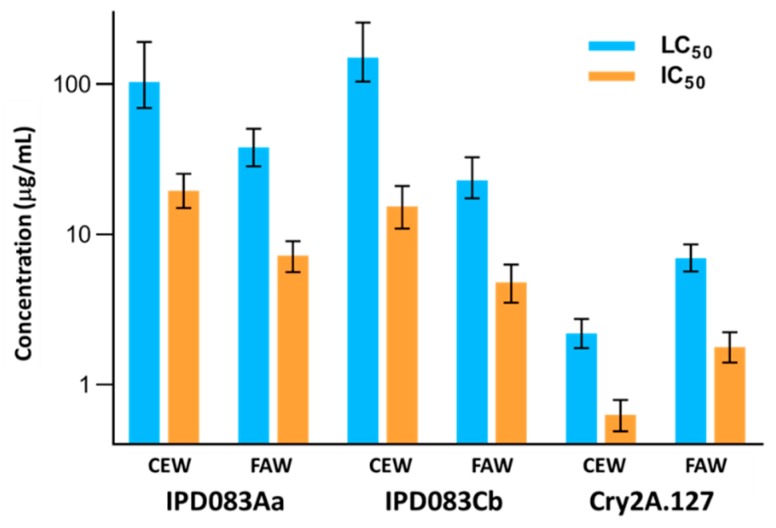
Evaluation of the insecticidal potency of the recombinant IPD083 proteins in artificial diet-based bioassay. The bioactivity of IPD083Aa, IPD083Cb, and Cry2A.127 are compared while Table S3 shows the complete set of data against CEW, FAW, SBL, and VBC. The IC_50_ is the concentration causing severe growth inhibition or death in 50% of the larvae and the LC_50_ is the concentration that caused 50% larval death (see Section 4.10). The bars reflect calculated LC_50_ or IC_50_ values and 95% fiducial limits are shown.

To facilitate characterization of the mechanism of insecticidal activity of the IPD083 proteins, we utilized a baculovirus-mediated insect cell expression system to produce recombinant proteins. Both His-tagged IPD083Aa and IPD083Cb recombinant proteins were purified using an Ni-NTA agarose column followed by an anion exchange polishing step. The isolated recombinant IPD083Aa and IPD083Cb showed high levels of purity (>95%) based on densitometry assessment of the SDS-PAGE gel image. Further characterization of the recombinant IPD083 proteins by size exclusion chromatography coupled with a multi-angle light scattering (MALS) detector showed that both proteins are in the monomer form (Figure S3). The insecticidal activities of purified IPD083Aa and IPD083Cb along with Cry2A.127 (an engineered Cry2Ab variant; see Section 4.12) proteins were evaluated on FAW, CEW, SBL, and VBC using artificial diet bioassays (Figure 5 and Table S3). The activities of the three proteins against SBL and VBC were comparable, while Cry2A.127 showed a higher potency on CEW and FAW. When assayed against a coleopteran insect, WCRW, at 150 µg/cm^2^ and a hemipteran insect, the southern green stinkbug (*Nezara viridula*), at 200 µg/mL, IPD083Aa and IPD083Cb showed no effect on either mortality or insect growth (Section 4.10). Although our ability to assess the activity spectrum on different orders of insect species was limited by the availability of insects, these results suggest that both IPD083Aa and IPD083Cb may have selective activity against lepidopteran insects.

### 2.7. Evaluation of the Interaction Between IPD083 Proteins and CEW or FAW Midgut Tissues

**Figure 6:**
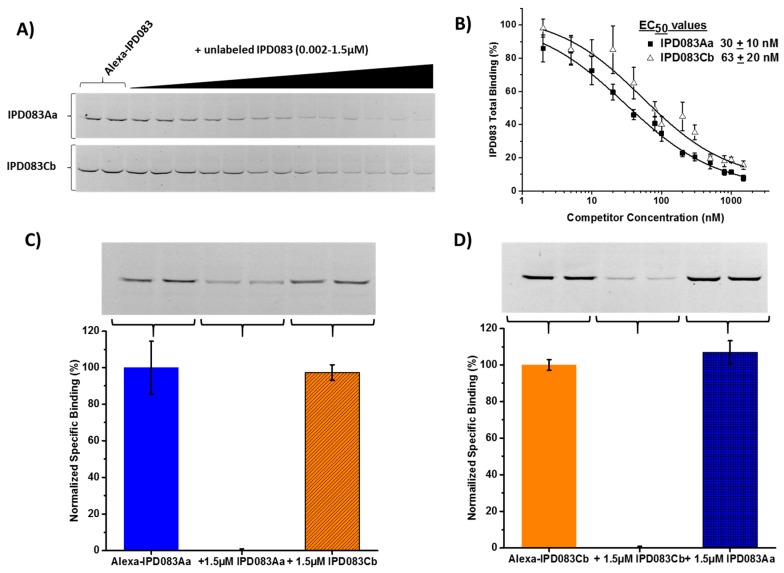
Assessment of the binding affinity and competition between IPD083Aa and IPD083Cb on CEW midgut tissue. (**A**) Gel images show concentration-dependent homologous competition between Alexa-labeled and unlabeled IPD083Aa or IPD083Cb. (**B**) Apparent binding affinity reported as EC_50_ determined from fitting the relationship of the signal from Alexa-labeled IPD083Aa or IPD083Cb vs. concentrations of their unlabeled counterparts. The Alexa-fluorescence signals were determined by densitometry performed on gel images, as represented in (**A**)**.** (**C**) A representative image of in-gel fluorescence is shown for competition between Alexa-IPD083Aa and unlabeled IPD083Aa (homologous competition) or IPD083Cb (heterologous competition). The fluorescence signal remaining in the presence of a homologous competitor reflects non-specific binding. Using densitometry software, normalized specific binding was calculated after subtraction of the remaining fluorescence signal (i.e., non-specific binding) in the presence of a homologous competitor (middle lanes of gel images). Each bar in the graph reflects the specific binding measured under each condition along with the standard error (see Section 4.11 for sample size and other details). (**D**) A representative image of in-gel fluorescence is shown for competition between Alexa-IPD083Cb and unlabeled IPD083Cb or IPD083Aa analyzed as described in (**C**).

We initiated the characterization of the mechanism of insecticidal activity of IPD083 proteins with CEW and FAW as representative insects. We employed brush border membrane vesicles (BBMVs) prepared from dissected insect midguts to assess the specific interaction of the IPD083 proteins in tissue that is known to be the target of Bt insecticidal proteins [37]. Specific binding to CEW BBMVs was demonstrated by the concentration-dependent competition of fluorescently-labeled IPD083Aa or IPD083Cb by its unlabeled counterpart. From those competition assays, we estimated the apparent affinities defined as the effective concentration for a 50% reduction in binding (EC_50_) of the labeled protein to CEW BBMVs, to be 30 nM for IPD083Aa and 63 nM for IPD083Cb (Figure 6A,B). These results suggest that the insecticidal activity of IPD083Aa and IPD083Cb likely involves a specific interaction with target sites in the CEW midgut similar to the interaction required for Bt protein toxicity [37].

**Figure 7:**
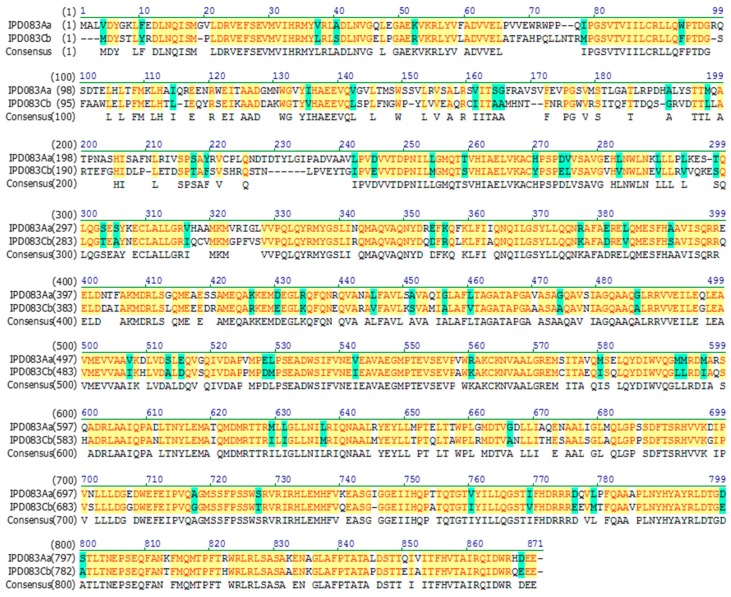
Protein sequence alignment of IPD083Aa and IPD083Cb. Alignment is done with a default set up (gap opening penalty: 10 and gap extension penalty: 01) of the Vector NTI program (Thermo Fisher Scientific). The N-terminal portion of the sequences shows a lower identity than that of the overall sequences.

Since IPD083Aa and IPD083Cb share approximately 71% protein sequence identity, it was important to determine whether IPD083Aa and IPD083Cb share the same target binding site(s). Heterologous competitive binding experiments were performed using CEW BBMVs (Figure 6C,D). No appreciable competition in either direction was observed between these two proteins, indicating that they recognize different CEW midgut target sites. Similar results were obtained when tested on BBMVs prepared from FAW (Figure S4B). A comparison of the protein sequences between IPD083Aa and IPD083Cb shows a lower level of identity within the first 330 amino acids (53%) and higher conservation for the rest of the proteins (82%) (Figure 7). Differences in midgut target sites might be explained by the less conserved N-terminal region, although further investigation will be required to understand the binding determinants for each of these proteins.

### 2.8. Evaluation of the Target Sites of IPD083 Proteins Against Major Classes of Lepidopteran Active Bt Proteins

**Figure 8:**
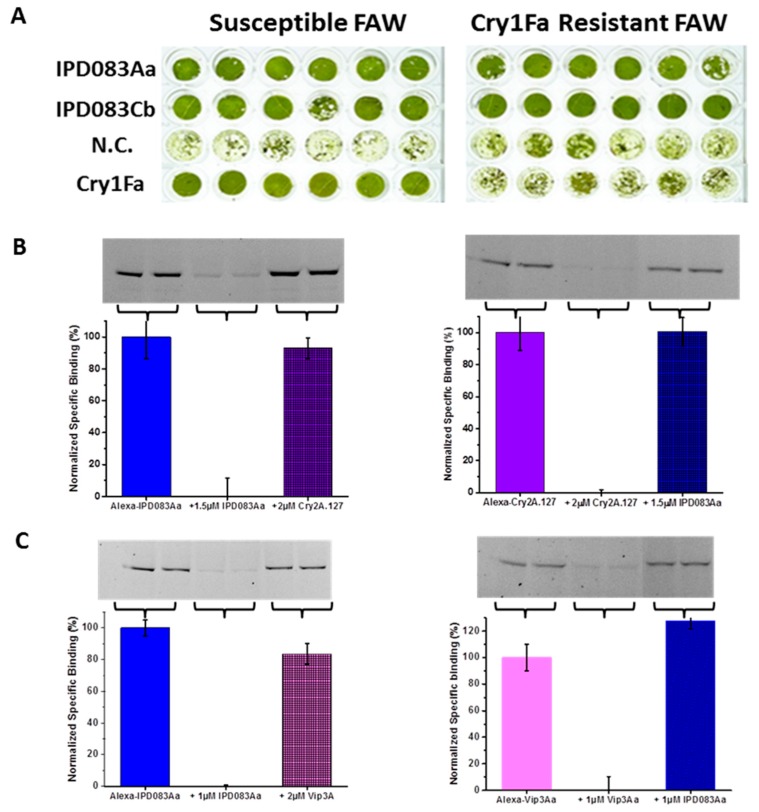
Evaluation of IPD083Aa and IPD083Cb for cross-resistance against classes of commercially deployed lepidopteran active Bt proteins. (**A**) Images of leaf disks transiently expressing IPD083Aa, IPD083Cb, Cry1Fa, or an empty vector (N.C.) after the infestation of either Cry1Fa-susceptible or -resistant fall armyworm (FAW) larvae. (**B**) Gel Images (upper panel) of reciprocal competition experiments between IPD083Aa and Cry2A.127 on CEW brush border membrane vesicles (BBMVs) and their corresponding quantitative analysis (lower panel) using densitometry. (**C**) Gel images (upper panel) of reciprocal competition experiments between IPD083Aa and Vip3Aa on CEW BBMVs and their corresponding quantitative analysis (lower panel) using densitometry. Normalized specific binding was calculated after subtraction of the remaining fluorescence signal (i.e., non-specific binding) in the presence of a homologous competitor (middle lanes of gel images). See Section 4.11 for details of sample size and data analysis. Reciprocal competition analyses between IPD083Cb and Cry2A.127 or Vip3Aa on CEW BBMV are provided in Figure S6.

High levels of resistance to Bt proteins can occur when there are changes in their target sites within the insect midgut [38]. Newly discovered insecticidal proteins are useful if their activity does not require binding to target sites that are utilized by Bt proteins currently in commercial products. To evaluate the potential for cross-resistance of IPD083Aa and IPD083Cb to major classes of lepidopteran active Bt proteins currently used in commercial transgenic crops, we utilized a Cry1Fa-resistant FAW strain derived from a field population collected in Puerto Rico [39] and BBMV-based heterologous competition binding assays for insect species where resistant colonies were not available to us. Common bean leaf disks transiently expressing IPD083 proteins were fed to Cry1Fa-susceptible or -resistant FAW strains to determine whether there were differences in leaf feeding damage between the two insect strains. The leaf disks expressing either the IPD083Aa or IPD083Cb protein showed near complete protection from feeding damage by both the Cry1Fa-susceptible and -resistant FAW. In contrast, leaf disks transiently expressing Cry1Fa were totally consumed by Cry1Fa-resistant FAW, while being protected against Cry1Fa-susceptible FAW, as expected (Figure 8A). Expression levels of Cry1Fa, IPD083Aa, and IPD083Cb were confirmed and estimated by semi-quantitative western blot analysis (Figure S5). We noticed that the expression levels for IPD083Aa and IPD083Cb proteins are roughly 16-fold and 11-fold higher, respectively, than that of Cry1Fa expressed under the control of the same promoter system. Overall, these results suggest that IPD083Aa and IPD083Cb control FAW with a target site(s) different from that utilized by Cry1Fa.

**Figure 9:**
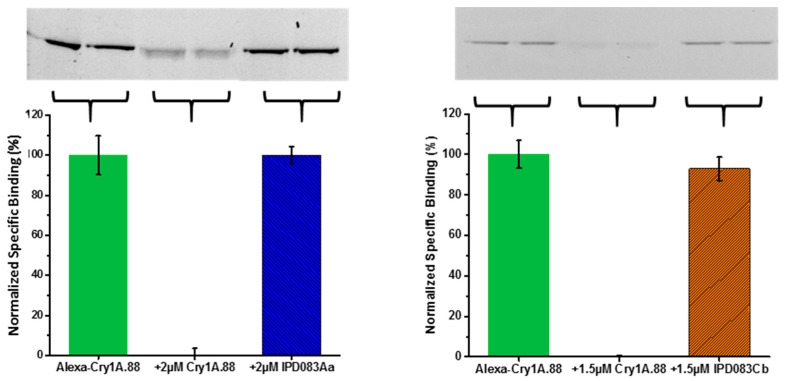
Heterologous competition binding between Cry1A.88 and IPD083 proteins on CEW BBMVs. Gel Images (upper panel) of competition between Alexa-Cry1A.88 and IPD083Aa (**left**) or IPD083Cb (**right**) and their corresponding quantitative analysis (lower panel) using densitometry. Normalized specific binding was calculated after subtraction of the remaining fluorescence signal (i.e., non-specific binding) in the presence of a homologous competitor (middle lanes of gel images). See Section 4.11 for details of sample size and data analysis.

With CEW and FAW as representative insects, we used recombinant IPD083Aa and IPD083Cb proteins to perform BBMV-based competitive binding assays against Vip3Aa and Cry2A.127. Heterologous competitive binding assays indicated no meaningful competition between IPD083Aa or IPD083Cb and Cry2A.127 or Vip3Aa on CEW BBMVs in either direction (Figure 8B, and Figure S6). In addition, no meaningful competition was observed between fluorescently-labeled Cry1A.88 (an engineered Cry1Ab variant; see Section 4.12) and unlabeled IPD083Aa or IPD083Cb, on CEW BBMVs (Figure 9). A panel of similar results was obtained from testing on BBMVs prepared from FAW (Figure S7). Competition binding assays with Cry1Fa were not performed as Cry1Fa was reported to share the same midgut binding sites with Cry1Ab in FAW BBMVs [40]. Our results indicate that IPD083Aa and IPD083Cb recognize different CEW or FAW midgut target sites from those recognized by several lepidopteran active Bt proteins currently used in commercial transgenic crops. Additional work is needed to understand the details of the interactions between the IPD083 proteins and the target insect midguts.

## 3. Discussion

Increasing global agricultural productivity is a critical need to meet the food supply demands of a growing world population for the next several decades. Our ability to effectively control insect pests of major crops plays a significant role in enhancing food security worldwide as insects account for losses of about 30% of pre-harvest and 10% of post-harvest yields [41]. In addition to controlling indigenous insect pests, the intercontinental introduction of highly damaging insect pests such as WCRW and FAW poses another challenge to increasing food production. Invasion of WCRW from North America to Europe, and more recently the introduction and the rapid spread of FAW originating from the Americas into the African and Asian continents are two examples that have had a major impact on corn production in those regions [13,14,15,42]. Since their introduction more than 20 years ago, crops developed with transgenic insect control traits have helped farmers to increase the yield and reduce the application of chemical pesticides [43]. Similar to the development of insect resistance to chemical pesticides, the emergence of insect resistance to some of the current commercial transgenic traits threatens the longer-term sustainability of such an effective pest management approach [8,9,10]. Current insect control transgenic traits have been developed with genes encoding Bt insecticidal proteins. However, the discovery of new Bt proteins with a desirable spectrum of activity which function as new modes of action (i.e., interacting with new target sites in the insect gut) is challenging and alternative sources are urgently needed.

The results reported here, along with other recent discoveries [19,20,21,22,23], further highlight the potential of non-Bt sources for new insecticidal proteins against various insect pests of major crops. The discovery of an insecticidal IPD083 protein family with variation in sequences, potencies, and activity spectra is of particular interest as it likely represents a new class of insecticidal proteins. Based on the common bean transient expression and leaf disk feeding assay results, IPD083Aa, IPD083Cc, and IPD083Cb showed favorable activity spectra for controlling major corn lepidopteran pests, including CEW, FAW, ECB, and BCW. IPD083Aa, IPD083Ca, IPD083Cb, IPD083Cc, IPD083Fh, and IPD083Fl all provided good protection against primary soybean pests, including CEW, SBL, and VBC. Insecticidal proteins with broad activity spectra against key pests offer advantages when used as pyramiding partners requiring a minimal number of co-expressed proteins for the reduced complexity of product development.

The observed level of CEW and FAW efficacy for transgenic corn and soybean plants expressing IPD083 proteins was surprising to us as their specific activities based on LC50/IC50 values are significantly lower than those of Cry2A.127 (Figure 5). Several different possible reasons for the observed efficacy include the fact that (1) protein stability in an artificial diet is different than in plants where the IPD083 protein is expected to be continuously expressed and actively accumulated; (2) high expression and/or accumulation levels of IPD083 proteins in plants may offset their lower specific activities; or (3) a higher percentage of IPD083 expressed in plants is properly folded than produced in a recombinant system, potentially resulting in an under estimation of the specific activities obtained from the artificial diet assay. The latter is quite likely as IPD083s were not able to be expressed as soluble proteins in *E. coli* and a low level of detergent was needed to effectively extract recombinant IPD083 proteins from the insect cell expression system (see Section 4.10). Studies are underway to quantify the expression and probe the in vivo efficacy of IPD083 proteins in transgenic corn and soybean plants.

While their mode of action is under further investigation, IPD083Aa and IPD083Cb have certain characteristics that are similar to Bt-derived proteins. Both IPD083 proteins are active orally against lepidopteran larvae and bind specifically to insect midgut-derived BBMVs, suggesting a toxic effect mediated via the insect digestive tract. The lack of activity against a coleopteran insect, WCRW, and an hemipteran insect, southern green stinkbug, under our testing conditions, suggests that IPD083 proteins may possess selectivity towards lepidopteran species. The ability of both IPD083 proteins to control Cry1Fa-resistant FAW and the lack of in vitro competition for binding when tested against several Bt proteins using CEW and FAW as representative insects suggest that IPD083 proteins likely recognize different CEW or FAW midgut target sites and therefore are unlikely to be cross-resistant to commercially deployed Bt proteins. This may not be surprising as none of the IPD083 family members share any known motifs, domains, or sequence signatures with other proteins. Although additional mechanistic studies are needed to understand the true MoA for those novel proteins, IPD083 proteins could be considered as effective pyramid partners with Bt proteins used in current commercial products or with any novel newly discovered insecticidal proteins to extend the durability of new transgenic insect control traits. Furthermore, IPD083Aa and IPD083Cb interact with different midgut target sites when compared to each other in both CEW and FAW BBMVs, which allows for the opportunity to deploy them as a pyramid in a resistance management strategy targeting key lepidopteran pests. As the utility of Bt proteins continues to be challenged by the adaptability of insect pests [8,9], IPD083Aa and IPD083Cb offer alternatives for the development of a new generation of transgenic insect control crops to benefit farmers in the battle against yield losses caused by lepidopteran pests.

## 4. Materials and Methods

### 4.1. Artificial Diet Insect Bioassays for Plant Diversity Screening and Protein Purification

CEW, FAW, VBC, and BCW eggs were obtained from Frontier Agricultural Sciences (Newark, DE, USA) and/or Benzon Research Inc. (Carlisle, PA, USA). ECB, SBL, WCRW, and southern green stink bug eggs were obtained from the DuPont Pioneer insectary (Johnston, IA, USA). Bioassays of CEW, ECB, FAW, and SBL were conducted using desalted protein extracts or purification fractions to evaluate the presence of insecticidal activity. For these initial screenings, 25 µL test samples were applied topically to the surface of 100 µL of a Lepidoptera-specific artificial diet (Southland Products Inc., Lake Village, AR, USA) in a 96-well plate, as described previously [22,44]. Assays were performed at 25 °C with two to three first instars per well and four replicates per sample. Assays of CEW and FAW were scored for insect mortality and inhibition of larval growth 3-days post infestation, while ECB and SBL, which feed less and develop slower, were scored 4-days post infestation.

### 4.2. Generation of Plant Transcriptome Sequence Libraries

Plants were sourced through various vendors within the USA. Total RNAs were isolated from frozen leaf tissues using the Qiagen^®^ RNeasy^®^ kit (Valencia, CA, USA). Sequencing libraries from the resulting total RNAs were prepared using the TruSeq™ mRNA-Seq kit and protocol from Illumina^®^, Inc. (San Diego, CA, USA). Sequencing was completed on the Genome Analyzer IIx, generating sixty million 75 bp paired end reads per normalized library. Paired end Illumina 75 bp reads were truncated at the first base with a quality score below 16, adapter sequences were trimmed, and reads shorter than 24 bp were eliminated using Flexbar2.4 [45]. Sequencing errors were corrected with SEECER (k = 19) v0.1.3 [46]. Reads with a median kmer (k = 31) abundance of less than 2 were removed with Khmer filter-abund.py v.0.4 and redundant reads were removed by the application of digital normalization targeting a maximum median kmer abundance of 25 with Khmer normalize-by-median.py v0.4 [47]. Transcript models were generated from the surviving reads using a panel of assembly software including Trinity r1011-11-26, Oases 0.2.01, IDBA-tran 1.1.1, and SOAPdenovo-Trans 1.03 [48,49,50,51]. Assemblies were generated across a range of kmer lengths from 23 to 51 in steps of 4 bp for those assemblers that accept multiple values of kmer. The final pool of candidate transcripts for a sample was derived by the filtration of redundant models using custom scripts to filter at decreasing levels of stringency for the nucleotide (100–97%) and predicted longest ORF (100–99%) sequences. All stop to stop ORFs with a length ≥25aa were generated for each transcript in the final set with the EMBOSS getorf tool [52].

### 4.3. Plant Sample Preparation, Protein Purification, and Identification

Crude extracts of diverse plant samples, including flowers, leaves, and tender stems, were prepared using the following protocol. Plant tissues were pulverized with 9.5 mm diameter steel balls at a liquid nitrogen temperature using a Geno/Grinder 2010 mill (Spex SamplePrep, Metuchen, NJ, USA) at 1600 RPM twice for 2 min. The frozen powder was extracted with 4 mL/g extraction buffer (100 mM Tris, pH 8, 150 mM KCl, 2.5 mM EDTA, 1.5% (*w*/*v*) polyvinylpolypyrrolidone, protease inhibitor cocktail (Roche, Indianapolis, IN, USA)) at 4 °C for 30 min and centrifuged at 18,000× *g* for 15 min. Supernatants were desalted using a Sephadex G25 column (GE Healthcare, Pittsburgh, PA, USA) and submitted for insect bioassays. For the purification of the IPD083Aa protein (Figure 1A), frozen sporulating fronds of *A. pedatum* were used to generate the crude lysate (note that sporulating fronds were the starting material for other Polypodiopsida as well). Instead of desalting, an equal volume of 70% (NH_4_)_2_SO_4_ in 20 mM Tris (pH 8) was added to the supernatant and after 2 h, incubation proteins were pelleted by centrifugation. Precipitated proteins were resuspended in 20 mM Tris, pH 8; clarified by centrifugation; and desalted using a Sephadex G25 column equilibrated in the same buffer. Eluted proteins were applied to a Mono Q column (GE Healthcare) which had previously been equilibrated in 20 mM Tris (pH 8). After the removal of unbound proteins, a linear 60 column volume (cv) gradient to 0.6 M NaCl in 20 mM Tris (pH 8) was applied and fractions with insecticidal activity were eluted at a conductivity of 24.6–30.5 mS/cm. These were buffer exchanged into 25 mM MOPS (pH 6.7) using a Sephadex G25 column and applied to a Mono P column (GE Healthcare) equilibrated in the same buffer. A 4 cv linear gradient to Polybuffer 74 (pH 4) (GE Healthcare) was applied and was followed by 15 cv Polybuffer 74 (pH 4). One fraction showed activity against SBL. Based on SDS-PAGE, the active fraction contained a protein band at approximately 90 kDa. The protein band of interest was excised and digested with trypsin after reduction and alkylation. The digest was analyzed by nanoLC-MS/MS on a Thermo Q Exactive Orbitrap mass spectrometer (Thermo Fisher Scientific, Waltham, MA, USA) interfaced with an Eksigent NanoLC Ultra 1-D Plus nano-lc system and a nanolc-as2 auto-sampler (AB Sciex, Framingham, MA, USA). The peptides were separated on a self-packed C18 reversed-phase capillary column (15 cm × 75 µm). Protein identification was performed by searching the LC-MS/MS data of tryptic peptides against an annotated protein database containing protein sequences translated from a transcriptome sequence library of the source plant using the Mascot search engine (Matrix Science, London, UK). For Mascot searches, the peptide mass tolerance was set at 5 ppm and the fragment mass tolerance was set at 0.2 Da.

### 4.4. Functional Verification of IPD083Aa and its Homologs in the Common Bean Transient Expression System

*IPD083Aa* was cloned using RT-PCR with *A. pedatum* total RNA preparation. cDNA was generated from 50 ng total RNA by reverse transcription with poly-dT oligo using the SuperScript^®^ First-Strand Synthesis System for RT-PCR (Invitrogen, Carlsbad, CA, USA) according to the manufacturer’s protocols. The gene encoding *IPD083Aa* was PCR amplified from its respective cDNA sample using primers designed for its coding sequence (Table S4) with Phusion^®^ High-Fidelity DNA Polymerase (New England Biolabs, Ipswich, MA, USA). The PCR product was then sub-cloned into a plant transient vector containing the DMMV promoter and a transcriptional terminator with a kanamycin selectable marker (NPTIII) [45]. The cloned *IPD083Aa* PCR product was confirmed by DNA sequencing. A transient expression vector containing the *IPD083Aa* gene and a vector backbone as the negative control were transformed into *Agrobacterium* cells. Leaves of common bean plantlets were infiltrated with the transformed cell culture as previously described [26,27]. Leaf disks were generated and pooled from common bean plantlets 7 days after infiltration with *Agrobacterium*. Eight randomly picked leaf disks (0.5 cm^2^) were infested with two to three neonates of FAW, ECB, and SBL. The images of leaf disks were recorded 3 days after infestation and insect mortality or growth inhibition was scored (Figure 2A). Four leaf disks expressing IPD083Aa were mixed with 200 µL ABCT-PI buffer (50 mM ammonium bicarbonate, 0.05% Tween20, protease inhibitor cocktail). Crude lysate was generated using a Geno/Grinder 2010 mill at 1600 RPM for 2 min with two 4 mm diameter steel balls. Soluble supernatant was obtained by centrifugation at 14,000× *g*, 4 °C for 30 min. Supernatant was separated on SDS-PAGE gel and a gel band corresponding to the 90 kDa region was excised (Figure 2B). Steps of in gel digestion and LC-MS/MS-based protein identification followed the same procedures as described in the protein purification section.

The NCBI GenBank accession numbers of the other selected IPD083 protein family members are listed in Table 1. Except for homologs *IPD083Fl* and *IPD083Fz* that were constructed by gene synthesis based on the transcriptome sequences, all others were cloned with a procedure similar to that of *IPD083Aa* using primers designed for their coding sequence (Table S4). Six randomly picked leaf disks (0.5 cm^2^) were infested with two to three neonates of each of the six lepidopteran species tested. The percentage of leaf disk consumption was visually estimated 3 days after infestation (Figure 3).

### 4.5. Vector Construction, Transformation, and Greenhouse Efficacy Evaluation for Soybean

Plasmid vector GmIPD083Aa was constructed containing the *Arabidopsis* Ubiquitin 10 promoter (AtUBQ10) [32] to drive the expression of *IPD083Aa* along with the *Arabidopsis* UBQ14 terminator [53]. Biolistic-based transformation into the TB5 target line and FLP-FRT-mediated RMCE using the GmIPD083Aa vector was performed as previously described [31]. Quantitative PCR (qPCR) was used to estimate the copy number of the expression cassette in transgenic T0 events and zygosity of T1 plants [33,54]. Single copy events estimated by qPCR were used for efficacy testing. Leaf disks (2 cm^2^) were collected from the V3 leaf of V4 T1 homozygous plants of the GmIPD083Aa construct, as well as non-transgenic plants (negative control), to compare leaf disk protection. Four leaf disks per plant from a total of 10 T1 plants per insect (total of 40 leaf disks for each of SBL, VBC, CEW, and FAW) were placed in 24-well plates on water-agar (2%) and each disk was infested with two neonates (0–24 h old). Infested plates were incubated in a growth chamber at 28 °C and 70–80% relative humidity. Leaf damage was assessed at 5 days post infestation using a Canon EOS 5D Mark II camera and DSLR Remote Pro for Windows (Breeze Systems, Surrey, UK) Version 3.1 and an internally developed image analysis program to assess the percentage of leaf damage compared to negative controls. Leaf damage data were subjected to analysis of variance and the means were compared by ANOVA (*p* < 0.0001) using the SAS data analysis package (SAS Institute, Cary, NC, USA). To detect IPD083Aa accumulation, four 6.3 mm diameter leaf disks per plant were lyophilized, pulverized, and resuspended in 200 µL phosphate buffered saline with Tween 20 (PBST) containing a Roche proteinase inhibitor cocktail. The samples were sonicated for 2 min and then centrifuged at 4 °C, 13,000× *g* for 15 min. Supernatants were mixed with Novex SDS-PAGE LDS loading buffer (Thermo Fisher Scientific, Waltham MA, USA), and run on Novex NuPAGE 4–12% Bis-Tris Midi gels with MES running buffer. Proteins were transferred onto a nitrocellulose membrane for 13 min using I-Blot apparatus (Thermo Fisher Scientific). After blocking with 5% skim milk powder in PBST, a purified rabbit anti-IPD083Aa primary antibody (Rockland Immunochemicals, Limerick, PA, USA) was used at 1:20,000, and a secondary goat anti-rabbit HRP conjugate was used at 1:20,000. Images were obtained on a Fujifilm imager after brief incubation in the presence of ECL™ Western Blotting Reagent (GE Healthcare) (Figure S1A).

### 4.6. Field Evaluation of Transgenic Soybean Plants Expressing the IPD083Aa Protein

In order to have sufficient seed for field testing experiments, a seed increase was performed using the T2 plant generation derived from efficacious T1 events. Three separate field experiments were conducted in Johnston, IA, USA in 2016 to evaluate the efficacy of IPD083Aa-expressing soybean T3 homozygous plants against three key lepidopteran species (SBL, VBC, and CEW) using manual infestation in screen cages. Experimental plots were planted on May 16, 2016. For each insect species, the experimental unit (20 T3 plants plus negative control) consisted of a single-row plot with a 76 cm length and row spacing of 91 cm. A randomized complete block experimental design was used with four replications for each species. Treatments consisted of two transformation events expressing IPD083Aa and the corresponding non-transgenic variety (DuPont Pioneer brand 93B86) as a negative control. A fungicidal seed treatment containing mefanoxam + fludioxonil was applied to all treatments at the labeled rate of 0.0092 mg ai/seed (ApronMAXX© Syngenta Crop Protection LLC, Greensboro, NC, USA). No foliar insecticide or herbicide was applied after soybean plant emergence. Manual infestation for each of the three lepidopteran species was achieved by releasing pupae. Pupae for SBL, VBC, and CEW were reared in the laboratory from eggs by the Corteva Agriscience Insect Production Research (IPR) group located in Johnston, IA, USA. Screen cages (8.8 m length, 4.9 m width, and 1.8 m height) were placed over each replication prior to infestation. The infestation rate for each experiment was 1000 pupae per tent (replicate) for SBL and VBC and 750 pupae per tent for CEW. Pupae were released inside cages when soybean attained the R2 growth stage [55] for all three lepidopteran species. Adult moths were allowed to emerge, mate, and lay eggs on soybeans. Visual ratings of percent defoliation to each plot were determined for each experiment 14 days after neonates emerged using previously described defoliation assessment methods [56] (http://www.extension.umn.edu/agriculture/soybean/pest/visual-guide-for-estimation-of-soybean-defoliation) (http://www.ipm.iastate.edu/ipm/icm/2002/7-29-2002/soydefoliation.html). A linear mixed model was used to analyze percent defoliation (Figure 4B).

Data for percent defoliation (*Y_imbns_*) of replication (*R*)*_i_*, treatment (*P*)*_m_*, background (*B*)*_b_*, construct (*E*)*_n_*, and plot *s*, were modeled as a function of an overall mean *μ*, factors for replication, treatment, background, treatment by background, construct, and a residual ɛ*_imbns_*. The model can be specified as

(1) $$Y_{imbns}=\mu +R_{i}+P_{m}+B_{b}+{\left(P\times B\;\right)}_{mb}+\;E_{n}+\varepsilon_{imbns}$$

where treatment was treated as a fixed effect, and all the other effects except for the residual were treated as independent normally distributed random variables with means of zero. For the residual factor, instead of assuming independence among plots, a two-dimensional separable first-order autoregressive correlation (AR1 × AR1) structure was applied to capture plot-to-plot correlations in both row and column directions of the field, besides the plot-to-plot variation. *F*-tests were used to assess the significance for fixed effects. Additionally, *t*-tests using standard errors from the model were conducted to compare treatment effects. A difference was considered statistically significant if the *p*-value of the difference was less than 0.05. All data analysis and comparisons were made in ASReml 3.0 (VSN International, Hemel Hempstead, UK, 2009).

### 4.7. Vector Construction, Transformation, and Greenhouse Efficacy Evaluation for Corn

Plasmid vector ZmIPD083Cb was constructed using the corn ubiquitin promoter (ZmUBI) [35] followed by the *IPD083Cb* gene and a terminator from *Solanum tuberosum* (PIN II) [47]. A plant selectable marker gene encoding phosphomannose isomerase (PMI) [57], driven by the ZmUBI promoter and its intron, was also present in the same vector. The corn transformation vector backbone was described by Komari et al. [58,59]. *Agrobacterium*-mediated stable corn transformation was performed using the described method [34]. Single copy events as estimated by qPCR were selected for subsequent insect control efficacy testing [33,54]. Leaf disks (1.13 cm^2^) from T0 hemizygous corn plants of the ZmIPD083Cb construct, positive control hemizygous plants expressing Cry1Fa (TC1507), and non-transgenic (negative control) plants were collected at the V6 stage from the youngest completely expanded leaf. Four leaf disks per plant of a total of 16 T0 events were collected for each insect assayed (total of 64 for each of ECB, CEW, and FAW) and placed on water-agar (2%) in 24-well plates. Neonate insects (3–7) (less than 24 h old) were placed on each leaf disk. Infested plates were incubated in a growth chamber at 27 °C and 70–80% relative humidity. Leaf damage was assessed at 24–48 h post infestation using a Canon EOS 5D Mark II camera and DSLR Remote Pro for Windows (Breeze Systems, Surrey, UK) Version 3.1 and an internally developed image analysis program to assess leaf damage compared to controls. IPD083Cb expression in corn leaves was detected by western blot analysis using a protocol similar to that for IPD083Aa expression in soy plants. Antibodies raised against IPD083Aa was used to visualize the expression of IPD083Cb as IPD083Cb specific antibodies were not available at the time the experiment was conducted (Figure S1B). Ear protection assays were performed for both CEW (with 9 ears) and FAW (with 7 ears) on a subset of T0 plants that demonstrated efficacy in leaf disk efficacy assays, as well as positive control plants expressing Cry1Fa (TC1507) (with 24 ears for CEW and 20 ears for FAW) and non-transgenic (negative control) plants (with 9 ears for CEW and 19 ears for FAW). Plants were artificially infested with either about 50 eggs (CEW) or 10 neonate insects (0–24 h old FAW) about 5–8 days after pollination. Eggs or neonates were infested at the ear tip with silks exposed and the ears were enclosed in a mesh bag to prevent larvae escape. Ear damage (in cm^2^) was measured about 2–3 weeks (CEW) or 2 weeks (FAW) post-infestation. Feeding damage greater than 15 cm^2^ was scored as 15 cm^2^ (maximum damage). For an ear damage comparison of IPD083Cb, positive and negative controls were subjected to analysis of variance and the means were compared by ANOVA (*p* > 0.0001) using the SAS data analysis package (SAS Institute, Cary, NC, USA).

### 4.8. Field Evaluation of Transgenic Corn Plants Expressing the IPD083Cb Protein

For evaluation in the field, second-generation (T2) non-segregating hybrid maize seeds, derived from eight original T0 transgenic events of the construct ZmIPD083Cb, were planted at three US locations, including Union City, TN; Sikeston, MO; and Waimea, HI, in 2017. Field trials consisted of single-row plots that ranged from 3.96 to 4.42 m in length, depending on the locations, and had rows spaced 76 cm apart (Table S2). A randomized complete block experiment design with three replications was utilized to arrange the treatments. Seeds in all treatments were treated with thiamethoxam (0.25 mg active ingredient/kernel), and no other insecticides were applied to the trial area. Plants were under natural infestation and evaluated at the R4 growth stage. CEW feeding injury was assessed on ears from five consecutive plants of each plot by measuring the total cm^2^ ear damage from the cob tip. A linear mixed model was applied to model the total cm^2^ of ear damage traits on individual plants for each location separately (Figure 4E and Table S2). Data for the total cm^2^ of ear damage trait (*Y_imnks_*) of the replication (*R*)*_i_*, construct (*P*)*_m_*, event (*E*)*_n_*, plot (*K*)*_k_*, and plant *s*, were modeled as a function of an overall mean *μ*, factors for replication, construct, event, plot, and a residual ɛ*_imnks_*. The model can be specified as

(2) $$Y_{imnks}=\mu +R_{i}+P_{m}+E_{n}+K_{k}+\varepsilon_{imnks}$$

where the construct was treated as a fixed effect, and all the other effects were treated as independent normally distributed random variables with means of zero. Additionally, *t*-tests were conducted to compare treatment effects. The statistical significance of differences, analyses, and comparisons was handled as described above for IPD083Aa expression in soy.

### 4.9. Test for FAW Cross-Resistance to IPD083Aa and IPD083Cb Caused by Selection With Cry1Fa

An FAW strain resistant to Cry1Fa [39] was used to evaluate cross-resistance to IPD083Aa and IPD083Cb in the common bean transient expression system and insect feeding bioassay. IPD083Aa, IPD083Cb, and Cry1Fa (GenBank accession number AAE8100) were cloned into the same transient expression vector as described in the section on functional verification of the IPD083 family members and an empty vector backbone was used as a negative control. Transient expression vectors were transformed into *Agrobacterium* cells. Common bean leaves were infiltrated with the transformed cell cultures as described. Leaf disks were generated and pooled from plantlets 3 days after *Agrobacterium* infiltration. Six randomly picked leaf disks were infested with two neonates of each of the Cry1Fa-susceptible and -resistant FAW strains. The degree of leaf disk consumption was visually evaluated and documented by digital photography 3 days after infestation (Figure 8). Pooled leaf disks, as three disks per sample, were analyzed by semi-quantitative western blot analysis using recombinant proteins as the standard and a corresponding polyclonal antibody raised against either IPD083Aa or IPD083Cb (Rockland Immunochemicals, Limerick, PA, USA). The expression level of each protein was estimated using densitometry and by comparison with a protein standard (Figure S5).

### 4.10. Recombinant Protein Production and Functional Verification of IPD083Aa and IPD083Cb

Recombinant IPD083Aa or IPD083Cb with a C-terminal 10X-histidine tag was expressed using the Bac-to-Bac Baculovirus Expression System (Invitrogen). The gene of *IPD083Aa* or *IPD083Cb* was inserted into the vector pFastBacDual. The plasmid was then transformed into DH10Bac1 cells to produce recombinant bacmid DNAs. Sf9 insect cells were transfected with bacmid DNA using Cellfectin for the baculovirus expression of IPD083Aa or IPD083Cb. Large-scale cultures of infected Sf9 cells were harvested 72 h post infection by centrifugation at 2000× *g* for 20 min. Insect cell pellets were resuspended with lysis buffer (137 mM NaCl, 2.7 mM KCl, 10 mM Phosphate, pH 7.4, 10% glycerol), plus a protease inhibitor cocktail (Roche, Indianapolis, IN, USA) and benzonase nuclease (Novagen, San Diego, CA, USA), and incubated at 4 °C for 30 min with stirring. The cells were lysed using a homogenizer (Constant Cell Disruption System, Warwickshire, UK) and zwitterionic surfactant CHAPS (MP Biomedicals, Solon, OH, USA) was then added to the lysate to produce a 1.0% final concentration and stirred at 4 °C for 1 h. The lysate was clarified by centrifugation at 30,000× *g* for 20 min. The resulting supernatant was loaded onto a Ni-NTA agarose column (Novagen) pre-equilibrated with lysis buffer. The column was then eluted with lysis buffer containing 10, 20, 50, and 250 mM of imidazole. The pool of partially purified fractions was loaded onto a 5 mL HiTrap Q HP column (GE Healthcare) and eluted with a 0 to 1 M NaCl salt gradient. The purified recombinant proteins were further characterized by size exclusion chromatography (SEC) coupled with a multi-angle light scattering (MALS) detector to assess the in-solution molecular weight [60]. An Agilent 1260 Infinity HPLC System equipped with a ProSEC 300s column (Agilent Technologies, Santa Clara, CA, USA) was linked to a miniDAWN Treos MALS detector (Wyatt Technologies, Santa Barbara, CA, USA). The protein was eluted from SEC with a PBS buffer (142 mM NaCl, 8.1 mM Na_2_HPO_4_, 1.5 mM KH_2_PO_4_, 2.7 mM KCl, pH 7.5) and was detected inline by the MALS detector. The molecular weight determination was performed using Astra software (Version. 6.1.2.84) (Wyatt Technologies). Protein purity was estimated by densitometry analysis of Coomassie blue stained SDS-PAGE and western blot analysis (Figure S3A,B). The purified recombinant proteins were further characterized by size exclusion chromatography (SEC) coupled with a multi-angle light scattering (MALS) detector to assess the in-solution molecular weight [60] (Figure S3C).

To verify the insecticidal activity of the purified recombinant IPD083Aa and IPD083Cb proteins, quantitative diet incorporation bioassays were conducted on CEW, FAW, SBL, and VBC [22,44] with a modification of using only 25% of the diet ingredients of the Southland Multiple Species Diet (Southland Products Incorporated, Lake Village, AK, USA). For CEW and FAW, each purified protein was tested at nine concentrations ranging from 1–208 μg/mL for IPD083Cb or 1–200 µg/mL for IPD083Aa. For SBL and VBC, each purified protein was tested at eleven concentrations ranging from 0.7–75 μg/mL. For all insects tested, bioassays were conducted in 96-well plates by mixing 25 µL of the respective test concentration with 35 µL of diet. Control insects were placed on a diet treated with Buffer (1x PBS that contained 10% glycerol). A single larva (< 16 h old first instar) was placed in each well. The assay was conducted for 32 replicates for each dose (*n* = 32 larvae). Assay plates were held in an incubator at 27 °C, 65 ± 5% RH. Insects were visually scored as dead, severely stunted (>60% reduction in size compared to control larvae), or not affected five days after infestation. The total numbers of dead and severely stunted larvae were used to calculate the growth inhibition concentration affecting 50% of the larvae (IC_50_) and the total numbers of dead larvae were used to calculate the lethal concentration affecting 50% of the larvae (LC_50_) (Table S3). Data for each protein bioassay were analyzed using the PROC PROBIT procedure employing the C = option in SAS software (2013, SAS Enterprise Guide 6.1, SAS Institute, Cary, NC, USA). To assess if IPD083Aa and IPD083Cb affect the growth of WCRW (coleopteran), we used a previously described method [22] with a modification of using diet overlay instead of incorporation in 96-well plates. Two doses (150 µg/cm^2^ and 75 µg/cm^2^) of IPD083Aa or IPD083Cb were tested and buffer was used as a negative control. The assay was conducted for 32 replicates for each dose (*n* = 32 neonates). The assay was scored for insect mortality and larval growth inhibition 5 days after infestation. To test if IPD083Aa and IPD083Cb affect the growth of southern green stinkbug (hemipteran), a diet bioassay was conducted using a single dose (200 µg/mL) of purified IPD083Aa or IPD083Cb protein in a total of 200 µL semi-solid insect diet (Bio-Serv F9644B, Frenchtown, NJ, USA) packaged into a small Parafilm (Bemis NA, Neenah, WI, USA) pillow pouch. Buffer was used as a negative control. Each pouch was placed in a small petri dish and infested with 5 s instar nymphs. For each sample, eight replicates were tested. The original pouch was replaced with a new one after 3 days and the assay was scored 6 days after the initial infestation.

### 4.11. Insect Brush Border Membrane Vesicle (BBMV) Binding of IPD083 Proteins and Competition Binding Studies

Insect midguts were isolated from the fourth instar insects by making a longitudinal incision through the cuticle along the dorsal side of the larvae. Midguts were opened to remove the peritrophic membrane and food bolus and separated from the carcass. Isolated guts were cleaned of fat bodies and other non-midgut tissues and immediately flash frozen in liquid nitrogen for storage at -80°C until needed. BBMVs were prepared from stored midgut tissue essentially as described in [61]. Protein levels were quantified by the colorimetric bicinchoninic acid (BCA) method (Pierce, Rockland, IL, USA). BBMV quantities for these studies were based on these protein determinations. Aminopeptidase N activity was measured and used as an indicator of apical membrane enrichment during BBMV preparation. Enrichment values for FAW and CEW BBMV were typically 5–6 and 4–9, respectively. In preparation for binding and competition, IPD083Aa and IPD083Cb proteins were dialyzed overnight at 4 °C in binding buffer which consisted of 20 mM Na_2_CO_3_, 20 mM NaHCO_3_, pH 9.6, 100 mM NaCl, and 0.2% Tween-20^®^. Cry2A.127, Vip3Aa, and Cry1A.88 proteins were treated with trypsin to simulate insect midgut processing, leaving stable core fragments that were purified by anion exchange column chromatography (HiTrap^TM^ Q FF 1 mL; GE Healthcare). Selected fractions were then combined and dialyzed into binding buffer. To track specific binding, each protein was labeled with the fluorescent indicator Alexa Fluor^®^488 (Thermo Fisher Scientific) according to the manufacturer’s instructions. Conditions for measuring specific binding were optimized by varying the amount of BBMVs and concentration of labeled protein while testing the competition caused by a molar excess of unlabeled protein. Optimal conditions were determined for each protein/insect combination as follows: 10 nM Alexa-IPD083Aa and 30 µg CEW BBMVs; 4 nM Alexa-IPD083Aa and 20 µg FAW BBMVs; 20 nM Alexa-IPD083Cb and 30 µg CEW or FAW BBMVs. Binding assays consisted of mixing an optimal amount of BBMVs and labeled protein in 100 μL of binding buffer in the absence and presence of different concentrations of unlabeled protein. The mixtures were incubated at 25 °C for 1 h while maintaining constant agitation using a high-velocity orbital shaker. Following the incubation, ice cold binding buffer (1.0 mL) was added to each reaction and the BBMVs with bound proteins were collected by centrifugation (10 min at 20,000× *g*) at 4 °C to separate unbound proteins. The BBMV pellet was then washed again with 1.0 mL of ice cold binding buffer with centrifugation (10 min, 20,000× *g*). The final BBMV pellet was then suspended in LDS sample buffer with reducing reagent (Invitrogen), boiled for 5 min, and subjected to SDS-PAGE (Novex NuPage^®^ 4–12% Bis-Tris gel using MOPS running buffer (Thermo Fisher Scientific). Upon the completion of electrophoresis, Alexa-labeled toxins were detected as fluorescent bands within the gels. The gel image was captured electronically using a digital imaging system (LAS-4010, GE Healthcare). Densitometry measurements of gel images were used to quantify the binding of the labeled toxins using image analysis software (TotalLab, Newcastle, UK). The EC_50_ values were measured by incubating BBMVs with the labeled protein in the absence and presence of increasing concentrations of unlabeled protein (homologous competition) until maximal reduction was achieved. For EC_50_ determinations, the densitometry values were normalized to the averaged fluorescence signal measured in the absence of a competitor (two determinations per experiment) and then reported as the mean and standard error of the mean (SEM) from three independent experiments consisting of a single determination for each concentration of competitor. Averaged normalized densitometry values were fit to a logistic equation using OriginPro 2015 (Originlab, Northampton, MA, USA) to determine the point of 50% reduction. For heterologous competition experiments, optimal binding conditions for the labeled protein were used and were tested at a saturating concentration of the competing unlabeled protein. Specific binding was determined by subtracting the nonspecific binding signal that remained with homologous competition (using a saturating concentration of unlabeled protein) from all values. These values were then averaged and normalized to the averaged value measured in the absence of a competitor. The data in bar graphs represent the mean and SEM from two to three independent experiments consisting of two to three determinations per condition.

### 4.12. Recombinant Bt Protein Production for Binding Studies

Cry2A.127 is a variant of Cry2Ab (GenBank accession number WP_001089638) [62] with substitutions at nine amino acid positions (K36R, K99Q, N106D, D240G, M241T, V324G, T575I, L611F, and I622T). It was expressed in *E. coli* BL21 (DE3) Star cells using a modified pMAL expression vector (New England Biolabs) that contained an N-terminal six-histidine maltose binding protein purification tag. Cell pellets were suspended in 50 mM Tris pH 8.0, benzonase and lysed using a cell homogenizer (Constant Cell Disruption System). Cry2A.127 was purified using an Ni-NTA affinity column. Elution of the MBP-free protein was achieved using thrombin (Calbiochem, San Diego, CA, USA) (2.5 U/mg Cry2A.127 fusion protein) in 100 mM ammonium carbonate. The eluted protein was filtered and immediately subjected to ion exchange chromatography (HiLoad 16/10 Q Sepharose HP column, GE Healthcare) to remove thrombin. The Cry2A.127-containing fractions were pooled and dialyzed into 20 mM sodium carbonate (pH 11). The purified Cry2A.127 protein was confirmed to be cross-resistant to a Cry2Ab cabbage looper- (*Trichoplusia ni*) resistant strain [63], indicating that these mutations did not alter the binding of the target site(s). Vip3Aa (GenBank accession number L48811) was expressed in *E. coli* BL21(DE3) Star cells, using a pET-21b expression vector (Qiagen) that contained an N-terminal poly-histidine purification tag and was purified by an Ni-NTA affinity column. Thrombin was added to cleave the protein from the tag and was removed by ion exchange chromatography similar to the Cry2A.127 purification scheme. The final Vip3Aa protein was dialyzed into 50 mM ammonium bicarbonate. Cry1A.88 is an engineered variant of the Cry1Ab protein with 13 amino acid substitutions, as previously described [64,65]. The Cry1A.88 protein was produced from an acrystalliferous Bt G8 strain as previously detailed [65]. The purified Cry1A.88 protein was confirmed to be cross-resistant to a Cry1Ab ECB-resistant strain [66], indicating that these mutations did not alter the binding of the target site(s).

## Figures and Tables

**Table 1:** Information on the selected IPD083 family members identified from different plant species.

Gene Name	Protein Sequence ID to IPD083Aa (%)	GenBank Accession Number	Organism
*IPD083Aa*	100	KY588369	*Adiantum pedatum L.*
*IPD083Ca*	70	KY588372	*Adiantum trapeziforme*
*IPD083Cc*	70	KY588373	*Adiantum trapeziforme*
*IPD083Cb*	71	KY588370	*Adiantum trapeziforme*
*IPD083Ci*	71	KY588371	*Rumohra adiantiformis*
*IPD083Ch*	74	KY588375	*Polystichum tsus-simense*
*IPD083Cf*	77	KY588374	*Lygodium flexuosum*
*IPD083Cu*	72	KY588376	*Dryopteris intermedia*
*IPD083Cv*	74	KY588377	*Dryopteris intermedia*
*IPD083Fa*	48	KY588378	*Polypodium musifolium*
*IPD083Fl*	48	KY588379	*Davallia tyermanii*
*IPD083Fh*	46	KY588380	*Phyllitis scolopendium*
*IPD083Fah*	49	KY588383	*Coniogramme venusta*
*IPD083Fz*	45	KY588382	*Osmunda regalis*
*IPD083Gb*	38	KY588381	*Adiantum raddianum*

## Supplementary Materials

The following are available online at https://www.mdpi.com/2072-6651/11/7/383/s1: Figure S1: Detection of IPD083Aa or IPD083Cb protein expression in T1 soy bean or T0 corn leaves; Figure S2: Protection of corn plants expressing IPD083Cb against insect damage in the greenhouse; Figure S3: Characterization of the purified recombinant IPD083Aa and IPD083Cb proteins; Figure S4: IPD083Aa and IPD083Cb bind different targets in CEW and FAW midgut BBMVs; Figure S5: Protein expression determined by semi-quantitative western blot analysis; Figure S6: Reciprocal competition binding of IPD083Cb against Bt proteins on CEW midgut BBMVs; Figure S7: Heterologous competition binding of IPD083 proteins against classes of commercially-deployed lepidopteran active Bt proteins on FAW BBMVs; Table S1: Pairwise percent sequence identity of the selected IPD083 family members; Table S2: Information on corn field trial locations, design, and mean CEW ear damage from natural CEW infestations in 2017; Table S3: Insecticidal efficacy of the recombinant IPD083Aa and IPD083Cb from the baculovirus-mediated insect cell expression system; Table S4: Cloning primers of IPD083 family members.

## Data and Materials Availability

The materials reported in this paper may be subject to third party ownership and/or to governmental regulations. Availability of materials described in this manuscript to academic investigators for non-commercial research purposes under an applicable material transfer agreement will be subject to the requisite permission from any third-party owners of all or parts of the material and to governmental regulation considerations. Obtaining permission from third parties will be the responsibility of the requestor. DNA sequences of IPD083Aa and selected homologs were deposited in Genbank with accession numbers from KY558369-KY558383. The supplementary materials contain additional information and data. Correspondence for materials should be addressed to M.E.N.
