# Supplementary material for: Identification and Evaluations of Novel Insecticidal Proteins from Plants of the Class Polypodiopsida for Crop Protection against Key Lepidopteran Pests
Source: Toxins (Basel). 2019 Jul 1;11(7):383. doi: 10.3390/toxins11070383 (PMC6669613; doi:10.3390/toxins11070383)
Supplement: Supplementary file 1 [file toxins-11-00383-s001.pdf]

Supplementary Materials: Identification and Evaluations of Novel Insecticidal Proteins from Plants of the Class Polypodiopsida for Crop Protection against Key Lepidopteran Pests

Lu Liu †, Eric Schepers, Amy Lum, Janet Rice, Nasser Yalpani, Ryan Gerber, Nuria Jiménez-Juárez, Fikru Haile, Alejandra Pascual, Jennifer Barry, Xiuli Qi, Adane Kassa, Matthew J. Heckert, Weiping Xie, Changkui Ding, Jarred Oral, Minh Nguyen, James Le, Lisa Procyk, Scott H. Diehn, Virginia C. Crane, Howard Damude, Carol Pilcher, Russ Booth, Lu Liu †, Genhai Zhu, Timothy M. Nowatzki, Mark E. Nelson *, Albert L. Lu and Gusui Wu

† Different authors.

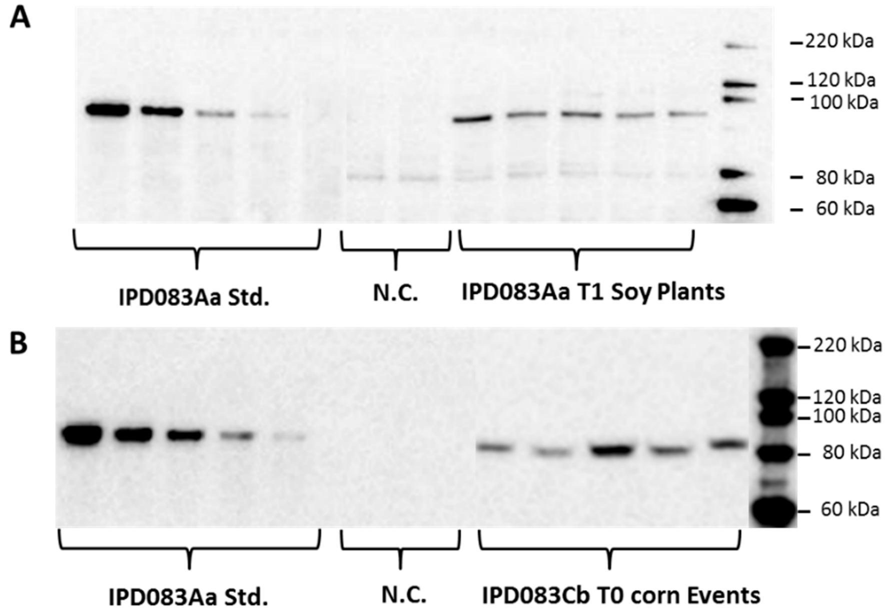

Figure S1. Detection of IPD083Aa or IPD083Cb protein expression in T1 soy bean or T0 corn leaves. (**A**) IPD083Aa protein expression was detected from soy bean leaves of five T1 plants of GmIPD083Aa construct showing feeding protection from various lepidopteran insects (Figure 3A). Negative control (N.C.) samples were from the soybean transformation line. (**B**) IPD083Cb protein expression was detected from corn leaves of five selected T0 events showing feeding protection from various lepidopteran insects (Figure 3B). Negative control (N.C.) samples were from the corn transformation line. Polyclonal antibody raised against IPD083Aa was used to visualize IPD083Cb as it cross-reacts to IPD083Cb. Protein standard for IPD083Cb was not available at the time this experiment was conducted.

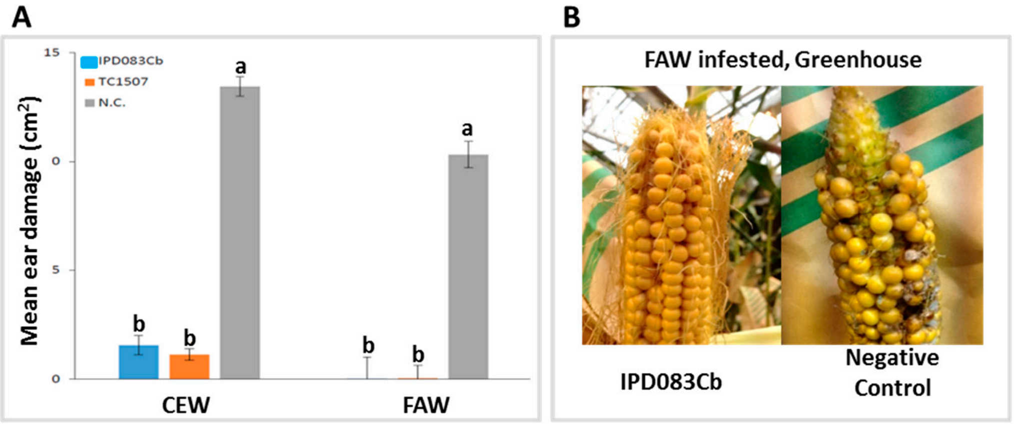

Figure S2. Protection of corn plants expressing IPD083Cb against insect damage in the greenhouse. (**A**) T0 transgenic corn plants, expressing IPD083Cb protein, exhibited ear protection in the greenhouse against CEW and FAW. Blue bars represent IPD083Cb expressing transgenic corn plants; orange bars represent Cry1Fa expressing corn plants (TC1507 event) as a positive control and gray bars represent non-transgenic control. Information on sample size is listed in the Main text Section 4.7. Results are presented as mean values with standard errors. Bars with different letters differ significantly at the $p < 0.05$ level (Tukey–Kramer test). (**B**) Corn ear images of transgenic plant expressing IPD083Cb (left) and non-transgenic negative control (right) after FAW infestation in the greenhouse.

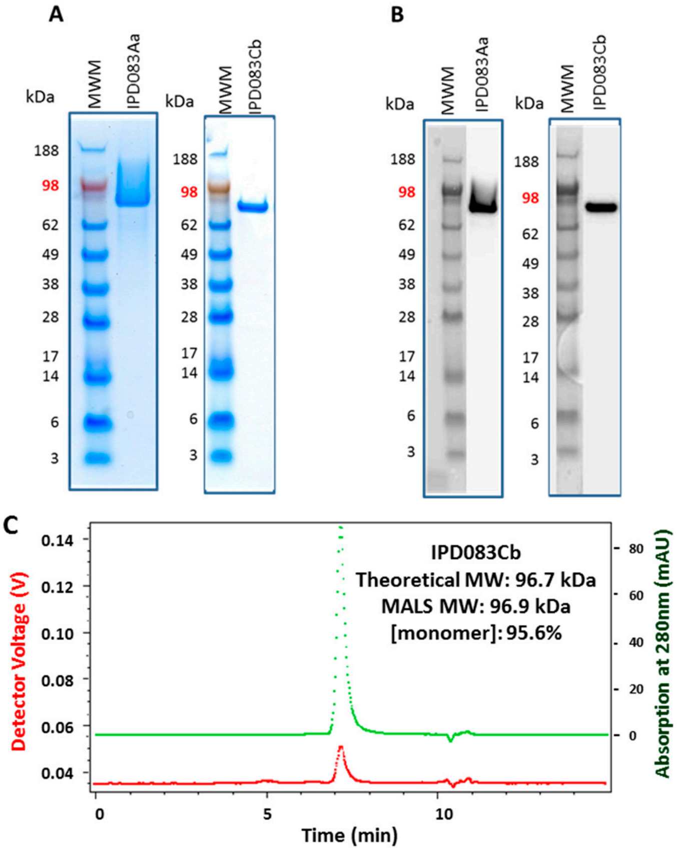

Figure S3. Characterization of the purified recombinant IPD083Aa and IPD083Cb proteins. The progress of purification was monitored by SDS-PAGE and (**A**) Coomassie blue staining or (**B**) Transferred to PVDF membrane and probed with mouse anti-His-tag antibody and goat HRP-mouse antibody. (**C**) SEC-MALS analysis showed that purified IPD083Cb is a monomer as the detected molecular weight matched closely to the theoretical one. Same result was obtained with purified IPD083Aa.

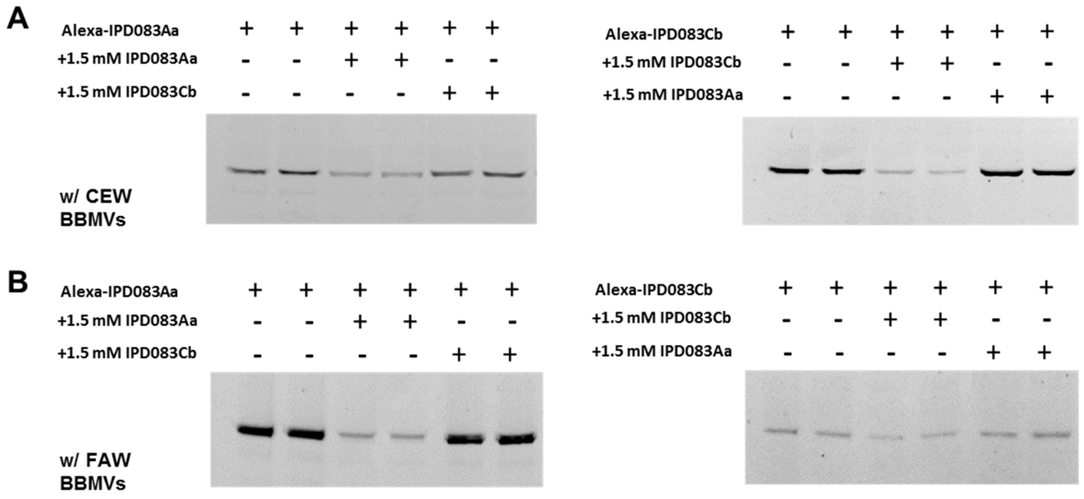

Figure S4. IPD083Aa and IPD083Cb bind different targets in CEW and FAW midgut BBMVs. Digital images of in-gel fluorescence reflecting **(A)** with CEW BBMVs (same gel images appearing in Main Figure 6C,D), specific binding of Alexa-IPD083Aa (or Cb) demonstrated by homologous competition by unlabeled IPD083Aa (or Cb) and the absence of competition by saturating concentrations of unlabeled IPD083Cb (or Aa) and (**B**) with FAW BBMVs, specific binding of Alexa-IPD083Aa (or Cb) demonstrated by homologous competition by unlabeled IPD083Aa (or Cb) and the absence of competition by saturating concentrations of unlabeled IPD083Cb (or Aa). The fluorescence signal remaining in the presence of homologous competitor reflects non-specific binding.

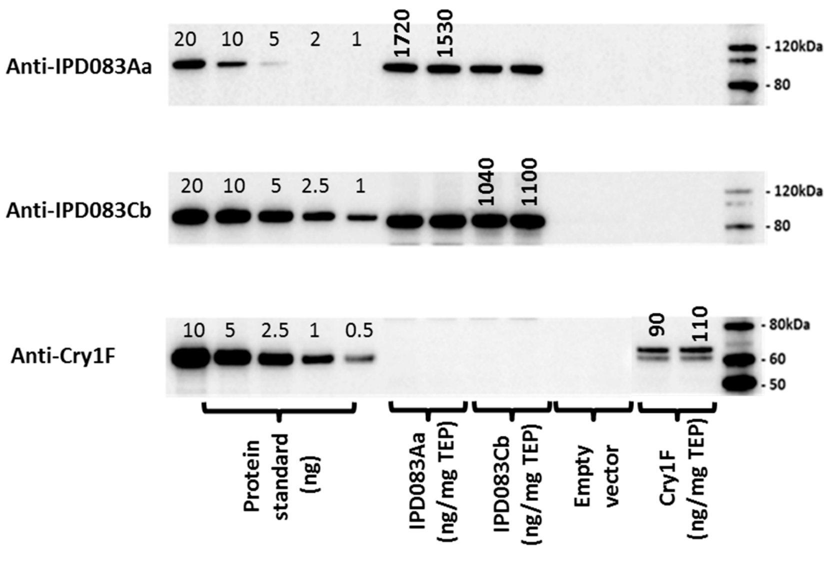

Figure S5. Protein expression determined by semi-quantitative western blot analysis. The corresponding leaf disk samples of Figure 8A were analyzed by pooling three disks as one single sample. Concentrations of protein standards are listed above gel bands and expression levels of IPD083Aa, IPD083Cb and Cry1Fa are estimated as ng/mg of total extracted protein (TEP). Polyclonal Antibody raised against IPD083Aa or IPD083Cb cross-reacts to both IPD083Aa and IPD083Cb proteins.

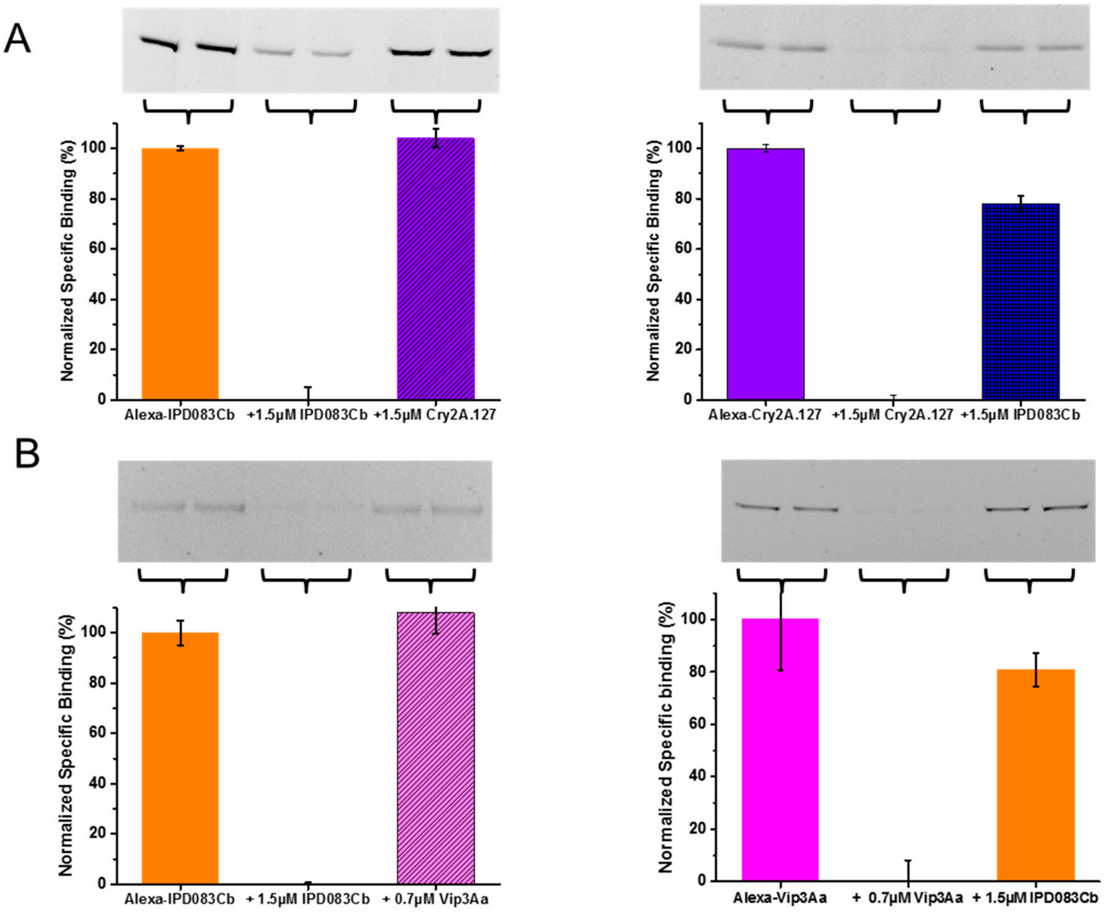

Figure S6. Reciprocal competition binding of IPD083Cb against Bt proteins on CEW midgut BBMVs. (**A**) Gel Images (upper panel) of reciprocal competition between IPD083Cb and Cry2A.127 and their corresponding quantitative analysis (lower panel) using densitometry. (**B**) Gel images (upper panel) of reciprocal competition between IPD083Cb and Vip3Aa and their corresponding quantitative analysis (lower panel) using densitometry. Normalized specific binding was calculated after subtraction of the remaining fluorescence signal (i.e., non-specific binding) in the presence of homologous competitor (middle lanes of gel images). See Main text Section 4.11 for details of sample size and data analysis.

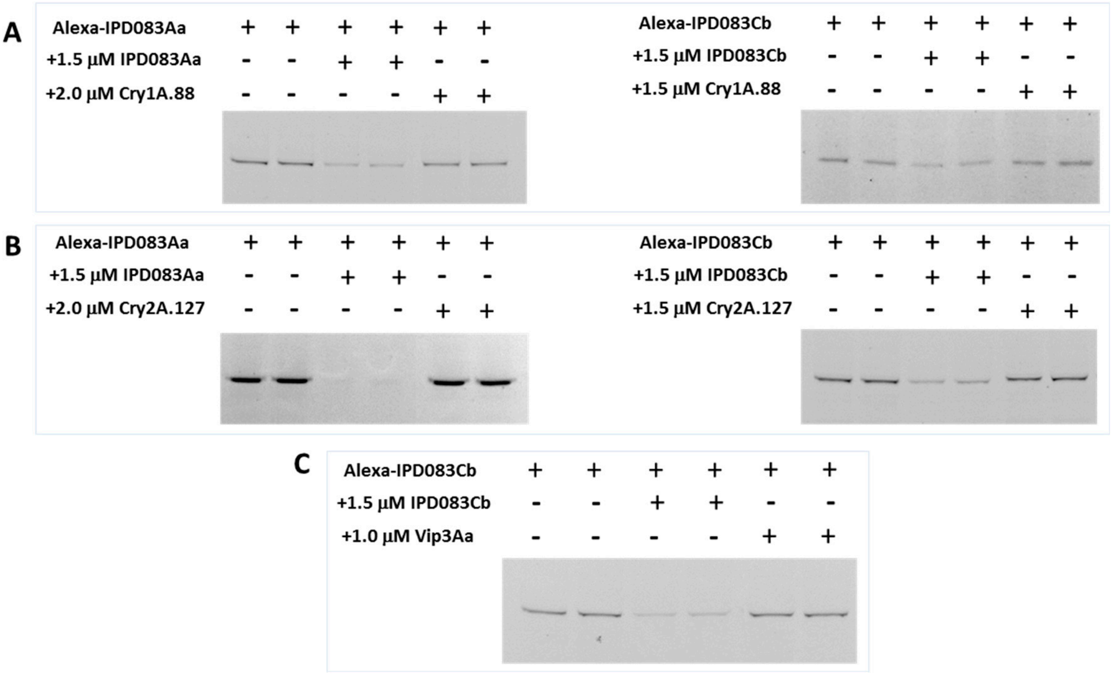

Figure S7. Heterologous competition binding of IPD083 proteins against classes of commercially deployed lepidopteran active Bt proteins on FAWBBMVs. (**A**) Gel images of competition between Alexa-IPD083Aa (left) or IPD083Cb (right) and Cry1A.88. (**B**) Gel images of competition between Alexa-IPD083Aa (left) or IPD083Cb (right) and Cry2A.127. (**C**) Gel images of competition between Alexa-IPD083Cb and Vip3Aa.

Table S1. Pairwise percent sequence identity of the selected IPD083 family members [a].

	IPD083Aa	IPD083Ca	IPD083Cc	IPD083Cb	IPD083Ci	IPD083Ch	IPD083Cf	IPD083Cu	IPD083Cv	IPD083Fa	IPD083Fl	IPD083Fh	IPD083Fah	IPD083Fz	IPD083Gb
**IPD083Aa**	100	70	70	71	71	74	77	72	74	48	48	46	49	45	38
**IPD083Ca**			98	76	75	80	79	71	73	47	47	44	47	43	37
**IPD083Cc**				76	75	80	79	72	73	47	47	45	47	43	37
**IPD083Cb**					97	78	77	71	71	48	48	46	47	44	38
**IPD083Ci**						77	77	71	71	48	48	46	48	44	39
**IPD083Ch**							84	75	77	49	49	47	48	45	40
**IPD083Cf**								79	80	48	48	47	50	46	39
**IPD083Cu**									94	49	49	46	50	46	40
**IPD083Cv**										49	49	47	50	46	40
**IPD083Fa**											91	63	45	40	38
**IPD083Fl**												62	45	39	38
**IPD083Fh**													44	40	37
**IPD083Fah**														43	38
**IPD083Fz**															47

[a] Pairwise percent sequence identity was performed with each pair of sequences using a default set of the Vector NTI sequence analysis software package (Thermo Fisher Scientific).

Table S2. Information on field trial locations, design, and mean CEW ear damage from natural CEW infestations in 2017.

Location	Plot Length (m)	Replications	Treatments	Mean CEW Ear Damage (cm^2) [a]	Standard Error
Waimea, HI	4.42	3	IPD083Cb	2.12 a	1.49
			Negative Control	30.90 b	2.68
Union City, TN	3.96	3	IPD083Cb	0.10 a	0.49
			Negative Control	10.45 b	0.96
Sikeston, MO	3.96	3	IPD083Cb	0.07 a	0.34
			Negative Control	5.34 b	0.68

[a] Best linear unbiased estimation (BLUE) was used to estimate mean treatment effects and values with different letters are significantly different ($p < 0.05$).

Table S3. Insecticidal efficacy of the recombinant IPD083Aa and IPD083Cb from the baculovirus-mediated insect cell expression system.

Toxin	Insect	LC_{50}/IC_{50} (μg/mL) [a]		95% FL	Slope	χ2 (df[b])	*P*-Value	*n*	NR[c] (%) (N) [d]
IPD083Aa	CEW	LC_{50}	103	69.4–190	1.35 ± 0.28	1.43 (3)	0.70	141	0.0 (30)
		IC_{50}	19.5	15–25.2	2.08 ± 0.25	5.36 (5)	0.37	202	0.0 (32)
	FAW	LC_{50}	38.0	28.3–50.4	1.89 ± 0.27	8.47 (5)	0.13	217	3.1 (32)
		IC_{50}	7.23	5.60–9.00	2.74 ± 0.39	2.35 (4)	0.67	185	3.1 (32)
	SBL	LC_{50}	2.19	1.60–2.90	1.28 ± 0.14	3.40 (7)	0.85	286	0.0(31)
		IC_{50}	0.25	0.19–0.33	1.81 ± 0.21	2.79 (6)	0.84	252	0.0 (31)
	VBC	LC_{50}	6.25	4.69–8.33	1.52 ± 0.16	6.52 (6)	0.37	251	0.0 (31)
		IC_{50}	1.30	0.49–2.28	2.47 ± 0.56	11.11 (4)[e]	0.03	187	0.0 (31)
IPD0083Cb	CEW	LC_{50}	150	104–257	1.89 ± 0.43	3.44 (3)	0.33	159	6.3 (32)
		IC_{50}	15.4	10.9–20.9	1.48 ± 0.20	4.95 (6)	0.55	255	6.3 (32)
	FAW	LC_{50}	22.9	17.4–32.6	1.88 ± 0.30	1.56 (3)	0.67	156	0.0 (32)
		IC_{50}	4.80	3.50–6.30	1.61 ± 0.18	3.61 (6)	0.73	242	0.0 (32)
	SBL	LC_{50}	0.47	0.31–0.68	1.19 ± 0.16	9.91 (6)	0.13	255	7.0 (29)
		IC_{50}	0.10	0.02–0.16	2.66 ± 0.70	8.54 (4)	0.07	191	10 (29)
	VBC	LC_{50}	0.23	0.19–0.28	3.18 ± 0.41	3.08 (3)	0.38	160	0.0 (31)
		IC_{50}	0.12	0.10–0.14	5.06 ± 0.82	0.03 (2)	0.99	128	0.0 (31)
Cry2A.127	CEW	LC_{50}	2.19	1.75–2.74	2.31 ± 0.25	3.82 (5)	0.58	222	0.0 (31)
		IC_{50}	0.63	0.49–0.79	2.22 ± 0.28	1.90 (4)	0.75	190	0.0 (31)
	FAW	LC_{50}	6.96	5.68–8.57	2.86 ± 0.37	4.19 (3)	0.24	157	0.0 (32)
		IC_{50}	1.78	1.40–2.23	2.33 ± 0.27	1.57 (5)	0.91	215	0.0 (32)
	SBL	LC_{50}	0.27	0.21–0.32	4.91 ± 1.05	2.07(3)	0.56	156	4.0 (27)
		IC_{50}	0.14	0.11–0.17	3.88 ± 0.68	3.79(3)	0.29	156	4.0 (27)
	VBC	LC_{50}	0.19	0.15 –0.24	2.43 ± 0.30	3.70(4)	0.45	185	0.0 (32)
		IC_{50}	0.10	0.08–0.13	2.43 ± 0.26	4.33 (6)	0.63	244	0.0 (32)

[a] LC_{50} is the concentration that caused 50% larval death; IC_{50} is the concentration causing severe growth inhibition or death in 50% of the larvae (see Main text Section 4.10); [b] *df* - Degrees of freedom; [c] NR - natural response as control mortality in percentage; [d] Number of control neonates; [d] Because of large chi-square ($p <$ 0.0254), a *t*-value of 2.78 was used in computing fiducial limits.

Table S4. Cloning primers of IPD083 family members.

Gene	Forward Primer	Reverse Primer
IPD083Aa	ccatggctctcgtggattacggcaag	gttaacctactcttcgtcgtgccgccagtc
IPD083Cb	cgaaatctctcatctaagaggctggatcctaggatggattacagcacgctttacagggac	ttaagttggccaatccagaagatggacaagtctagactactcctcctcttgccgccagtc
IPD083Ci	cgaaatctctcatctaagaggctggatcctaggatggattacagcactctttacacgg	taagttggccaatccagaagatggacaagtctagactactcctcttgccgccagtc
IPD083Ca	cgaaatctctcatctaagaggctggatcctaggatggattacagcacgctttacagggac	ttaagttggccaatccagaagatggacaagtctagactactcctcctcttgccgccagtc
IPD083Cc	cgaaatctctcatctaagaggctggatcctaggatggattacagcacgctttacagggac	ttaagttggccaatccagaagatggacaagtctagactactcctcctcttgccgccagtc
IPD083Cf	cgaaatctctcatctaagaggctggatcctaggatggccagtgtactggattacagcac	ttaagttggccaatccagaagatggacaagtctagactactcctcctcgtgccgcc
IPD083Ch	cgaaatctctcatctaagaggctggatcctaggatggattacagcacgctttacagg	taagttggccaatccagaagatggacaagtctagactactcctccacctcctgcctcc
IPD083Cu	cgaaatctctcatctaagaggctggatcctaggatggccgtcatggattacagcg	gttggccaatccagaagatggacaagtctagactactcgtcgtgccgccaatc
IPD083Cv	cgaaatctctcatctaagaggctggatcctaggatggccgtcatggattacagcg	gttggccaatccagaagatggacaagtctagactactcgtcgtgccgccaatc
IPD083Fa	tttaacttagcctaggatccatggaatatagcagcttgtac	actccttctttagttaacttactccacatcaccctcttgtcg
IPD083Fl	gene synthesized	
IPD083Fh	cgaaatctctcatctaagaggctggatcctaggatggagtactccgacttgtatgagg	taagttggccaatccagaagatggacaagtctagatcactcctcatcgacttcccg
IPD083Gb	cgaaatctctcatctaagaggctggatcctaggatggatgtggattatgcagccg	gttggccaatccagaagatggacaagtctagactaggtagtgctactagagtcgacacg
IPD083Fz	gene synthesized	
IPD083Fah	cgaaatctctcatctaagaggctggatcctaggatggctgctgcggcggagc	gttggccaatccagaagatggacaagtctagactagagagaaatccgcctgatagc
